# Clinically relevant mutations in the PhoR sensor kinase of host-adapted *Mycobacterium abscessus* isolates impact response to acidic pH and virulence

**DOI:** 10.1128/spectrum.01588-23

**Published:** 2023-10-24

**Authors:** Juan M. Belardinelli, Divya Arora, Charlotte Avanzi, William H. Wheat, Josephine M. Bryant, John S. Spencer, Tom L. Blundell, Julian Parkhill, R. Andres Floto, Mary Jackson

**Affiliations:** 1 Department of Microbiology, Immunology and Pathology, Mycobacteria Research Laboratories, Colorado State University, Fort Collins, Colorado, USA; 2 Department of Medicine, Molecular Immunity Unit, University of Cambridge, MRC-Laboratory of Molecular Biology, Cambridge, United Kingdom; 3 University of Cambridge Centre for AI in Medicine, Cambridge, United Kingdom; 4 Department of Biochemistry, University of Cambridge, Cambridge, United Kingdom; 5 Wellcome Sanger Institute, Hinxton, United Kingdom; 6 Department of Veterinary Medicine, University of Cambridge, Cambridge, United Kingdom; 7 Cambridge Centre for Lung Infection, Papworth Hospital, Cambridge, United Kingdom; Indian Institute of Science, Bangalore, Karnataka, India

**Keywords:** *Mycobacterium*, *abscessus*, redox homeostasis, acidic pH, phoPR, two-component response regulator

## Abstract

Functional genomics analysis of *Mycobacterium abscessus* clinical isolates from chronically infected patients to identify genes under strong evolutionary pressure during lung adaptation identified *phoR* as one of the most frequently mutated. *phoR* encodes the histidine kinase (HK) of the two-component regulatory system (TCS) PhoPR. While PhoPR has been extensively studied in *Mycobacterium tuberculosis* for its role in virulence, little is known about the function of this TCS and the signals governing its activation in *M. abscessus*. We here show that acidic pH leads to the upregulation of *phoP* in *M. abscessus* and that clinically relevant non-synonymous mutations identified in PhoR exacerbate this response. PhoR modulates the ability of its cognate response regulator, PhoP, to autoregulate itself by controlling its dephosphorylation. At low pH, the phosphatase activity of PhoR is reduced and the build-up of phospho-PhoP that ensues results in the upregulation of *phoP* that accompanies the induction of a defined set of genes, many of which are thought to play a role in virulence and host adaptation. In line with the idea that *M. abscessus* isolates expressing clinically relevant PhoR variants may be better prepared for survival within the host, we find these strains to be less efficiently internalized by macrophages and to display enhanced intracellular survival.

**IMPORTANCE:**

Difficult-to-treat pulmonary infections caused by nontuberculous mycobacteria of the *Mycobacterium abscessus* group have been steadily increasing in the USA and globally. Owing to the relatively recent recognition of *M. abscessus* as a human pathogen, basic and translational research to address critical gaps in diagnosis, treatment, and prevention of diseases caused by this microorganism has been lagging behind that of the better-known mycobacterial pathogen, *Mycobacterium tuberculosis*. To begin unraveling the molecular mechanisms of pathogenicity of *M. abscessus*, we here focus on the study of a two-component regulator known as PhoPR which we found to be under strong evolutionary pressure during human lung infection. We show that PhoPR is activated at acidic pH and serves to regulate a defined set of genes involved in host adaptation. Accordingly, clinical isolates from chronically infected human lungs tend to hyperactivate this regulator enabling *M. abscessus* to escape macrophage killing.

## INTRODUCTION

The *Mycobacterium abscessus* group (MABS) which is comprised of three subspecies, [*Mycobacterium abscessus* subsp. *abscessus* (*Mabs*), *Mycobacterium abscessus* subsp. *massiliense* (*Mmas*), and *Mycobacterium abscessus* subsp. *bolletii*]*,* is responsible for an increasing number of pulmonary infections, particularly in susceptible individuals with structural or functional lung conditions such as cystic fibrosis (CF), chronic obstructive pulmonary disease, and bronchiectasis. MABS infections cause accelerated inflammatory lung damage and are notoriously difficult to treat with antibiotics (1 – 3).

Understanding the complex physiological processes underlying the ability of MABS to become a chronic pathogen of the human lung could have an important impact on the development of prophylactic and therapeutic strategies to better control and treat MABS infections. One approach taken by our group and others toward this goal has focused on analyzing the whole genome sequence of serially isolated strains from CF and non-CF patients to gain insight into the genetic basis of host adaptation (4 – 8). The examination of longitudinal isolates from 201 CF patients identified an excess of non-synonymous single-nucleotide polymorphisms (SNPs) in a small subset of genes that are thus likely to be under strong evolutionary pressure (5). Of these genes, *phoR*, encoding the sensor kinase of the two-component regulatory system (TCS) PhoPR, was identified as the most common gene to acquire non-synonymous mutations during lung infection, with SNPs found in 15 out of 201 patients (5). Mutations in *phoR* were further highlighted in two other independent longitudinal studies (7, 9). Intriguingly, a similar analysis of genomic data from laparoscopy-associated MABS wound infections revealed that non-synonymous mutations in *phoR* occurred at significantly lower rates than observed during pulmonary disease suggesting that these mutations may specifically be important for lung infection (5). In support of an important role of *phoR* in host adaptation, an isogenic *Mmas* strain expressing a clinically relevant mutated form of PhoR displayed increased replication in human primary macrophages compared to the wild-type (WT) parent strain, and the same mutation promoted pathogenicity in a mouse model of pulmonary infection (5). Despite evidence for the contribution of PhoPR to lung adaptation, the precise function of this transcriptional regulator in MABS and the environmental cue(s) governing its activation or repression during infection remained undefined.

PhoPR is one of the best characterized TCS of *M. tuberculosis*. Similar to recent observations made in MABS, *phoR* mutations in tuberculous mycobacteria seem to have been under selection since the early spread of human tuberculosis and continue to occur during infection in current epidemiological settings (10). However, unlike the situation in MABS in which the genetic disruption of *phoPR* causes an enhancement of virulence (5), disruption of *phoP* or *phoPR* in *M. tuberculosis* (*Mtb*) dramatically attenuates virulence in macrophages and in mice (11 – 14). The virulence attenuation of *Mtb phoPR* knock-out (KO) mutants is explained by the critical role played by *Mtb* PhoP in the regulation of a number of functions relevant to intracellular adaptation. These include the control of bacterial replication and the maintenance of redox homeostasis at acidic pH and under hypoxia (15 – 20), the control of heat-shock protein and bioactive surface glycolipid production, and the modulation of the secretion of proteinaceous virulence factors, including ESAT-6 (11, 13, 21 – 23).

In this work, we show that acidic pH activates the PhoPR TCS of MABS leading to the upregulation of a defined set of genes, many of which are likely to play a role in virulence and the maintenance of redox homeostasis during infection. Clinically relevant non-synonymous SNPs in the periplasmic sensor loop of PhoR exacerbate this response leading to higher levels of expression of *phoP* and *phoP*-dependent genes at neutral pH (pH 7.0) and even greater levels of expression of the same genes relative to the WT parent strain when the pH drops to 5.7. The altered transcriptional response to pH of strains expressing patient-derived variants of PhoR enhances their ability to avoid macrophage killing.

## RESULTS

### PhoPR-dependent upregulation of *phoP* expression at acidic pH

In *M. tuberculosis*, the PhoPR TCS plays a critical role in the adaptation of the bacterium to become a chronic pathogen of the lung by allowing it to integrate a number of signals typifying, in particular, the phagosomal environment of macrophages (e.g., acidic pH and hypoxia) and subsequently adapt its metabolism to enhance survival (15 – 19). Whether PhoPR plays a similar role in MABS is unclear since the disruption of this regulator enhances rather than decreases virulence in this species (5). Moreover, prior transcriptomics studies failed to show an upregulation of MABS *phoPR* inside J774.2 macrophages despite evidence of *phoP* induction inside free-living amoebae (24).

**Fig 1:**
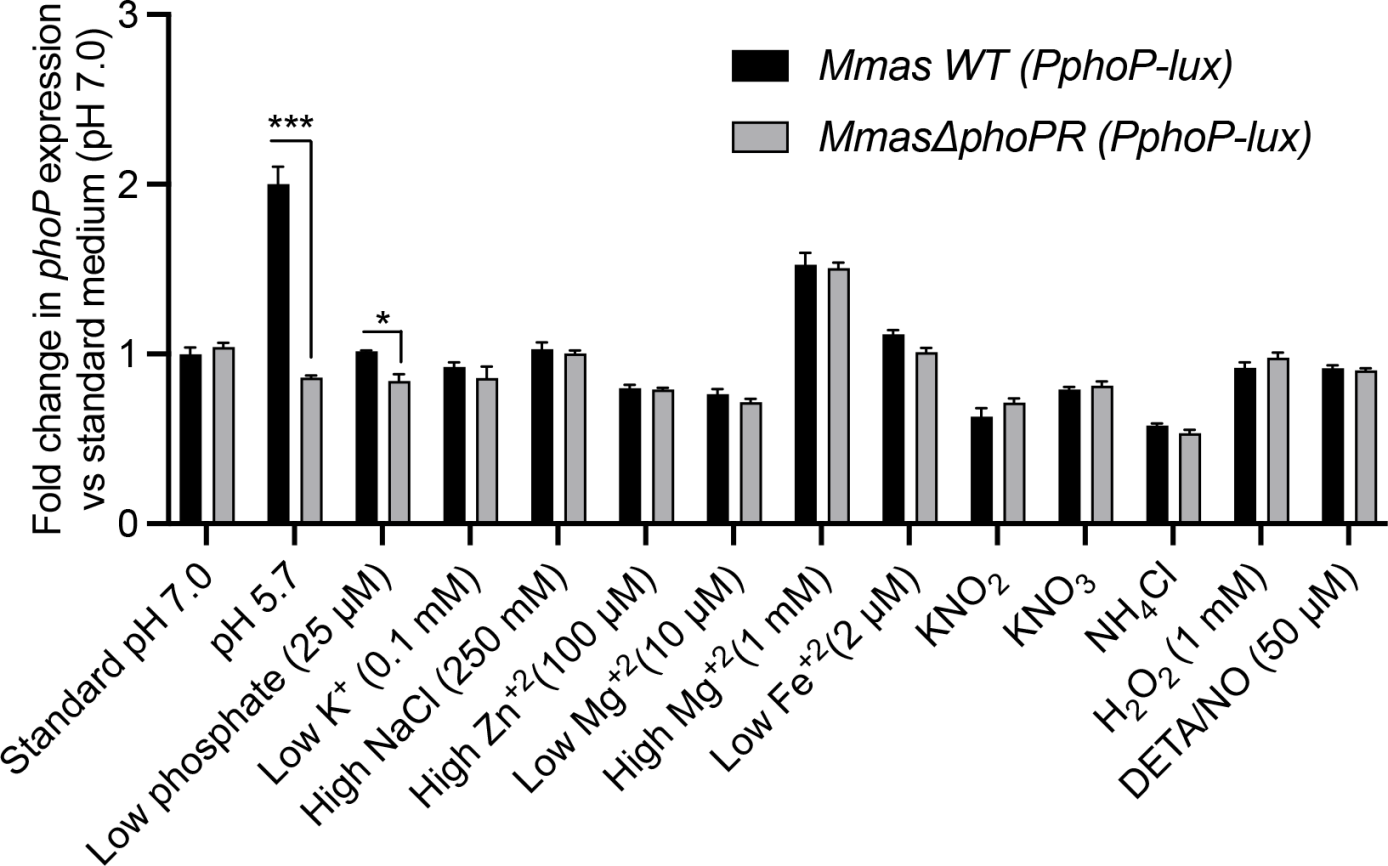
Acidic pH induces the expression of *phoP*. WT *Mmas* and *Mmas*Δ*phoPR* harboring a luciferase reporter gene under control of the *phoPmas* promoter (P*phoP-lux*) were grown in minimal medium at pH 7.0 (standard medium) or the same minimal medium modified to test different stresses as indicated on the *x*-axis. The luciferase activity of early log phase cultures (OD_600nm_ ~ 0.2–0.4) was measured and normalized to the OD_600nm_ of the culture. Fold changes in luciferase activity relative to the standard (pH 7.0) medium (arbitrarily set to 1) are shown. Assays were performed in triplicate and are representative of at least two independent experiments. Asterisks denote statistically significant differences between culture conditions pursuant to the unpaired Student’s *t* test (*P < 0.01, ***P < 0.0001).

In order to determine what stimuli may control the expression of the response regulator encoded by *phoP*, we fused 130 bp of the promoter region of *phoP* from *M. abscessus* subsp. *massiliense* CIP108297 (*Mmas*) to a luciferase reporter gene (*lux*). Both WT *Mmas* and the corresponding *phoPR* KO mutant, *Mmas*Δ*phoPR,* were transformed with the resulting plasmid and grown under a variety of conditions where luciferase expression was monitored. Growth in 7H9-ADC-Tween 80 medium indicated that *lux* expression was equally active in *Mmas* WT and *Mmas*Δ*phoPR,* and most pronounced during the early stages of growth, progressively decreasing from mid-log phase onward (Fig. S1). We next tested a variety of stresses known to activate PhoPR in *M. tuberculosis* and other prokaryotes, including acidic pH, phosphate starvation, low and high divalent cation concentrations, high chloride concentration, oxidative and nitrosative stresses, and growth in the presence of different nitrogen sources. The only condition under which *lux* expression was markedly (approximately twofold) induced in WT *Mmas* was at acidic pH (Fig. 1). This response was lost in *Mmas*Δ*phoPR* indicating that it is dependent on PhoP and/or PhoR (Fig. 1). Although *lux* expression increased with the concentration of magnesium in the medium, this regulation was independent of the presence of PhoP and PhoR since it was comparable in the WT strain and the *phoPR* deletion mutant (Fig. 1).

Since PhoPR was shown to be required to slow the growth of *M. tuberculosis* at acidic pH in the presence of certain carbon sources (16), we further sought to compare the growth and level of expression of *phoP* in the WT and *phoPR* KO strains cultured under acidic (pH 5.7) and neutral (pH 7.0) pH with either glucose, glycerol, pyruvate, or a combination of oleic acid and glucose as carbon sources. At both pHs, *Mmas* grew best in the presence of a combination of oleic acid and glucose, whereas pyruvate was the least preferred carbon source (Fig. S2A). In contrast to the situation in *M. tuberculosis*, *Mmas*Δ*phoPR* grew at a similar rate as WT *Mmas* whatever the carbon source at both neutral and acidic pH (Fig. S2A). However, in all media, luciferase expression from the *phoP* promoter was clearly induced in *Mmas* WT at pH = 5.7 relative to pH 7.0, whereas it remained unchanged in the *phoPR* mutant (Fig. S2B). Thus, independent of the carbon source, the expression of *phoP* is induced under acidic pH and PhoP and/or PhoR is(are) required for this induction to occur. Of note, the WT and *phoPR* mutant strains also displayed comparable growth rates under all other conditions tested in Fig. 1, except for a slight but reproducible impairment of the mutant’s growth when nitrite was used as the nitrogen source (Fig. S3).

### Phosphorylation enhances PhoP binding to its own promoter

**Fig 2:**
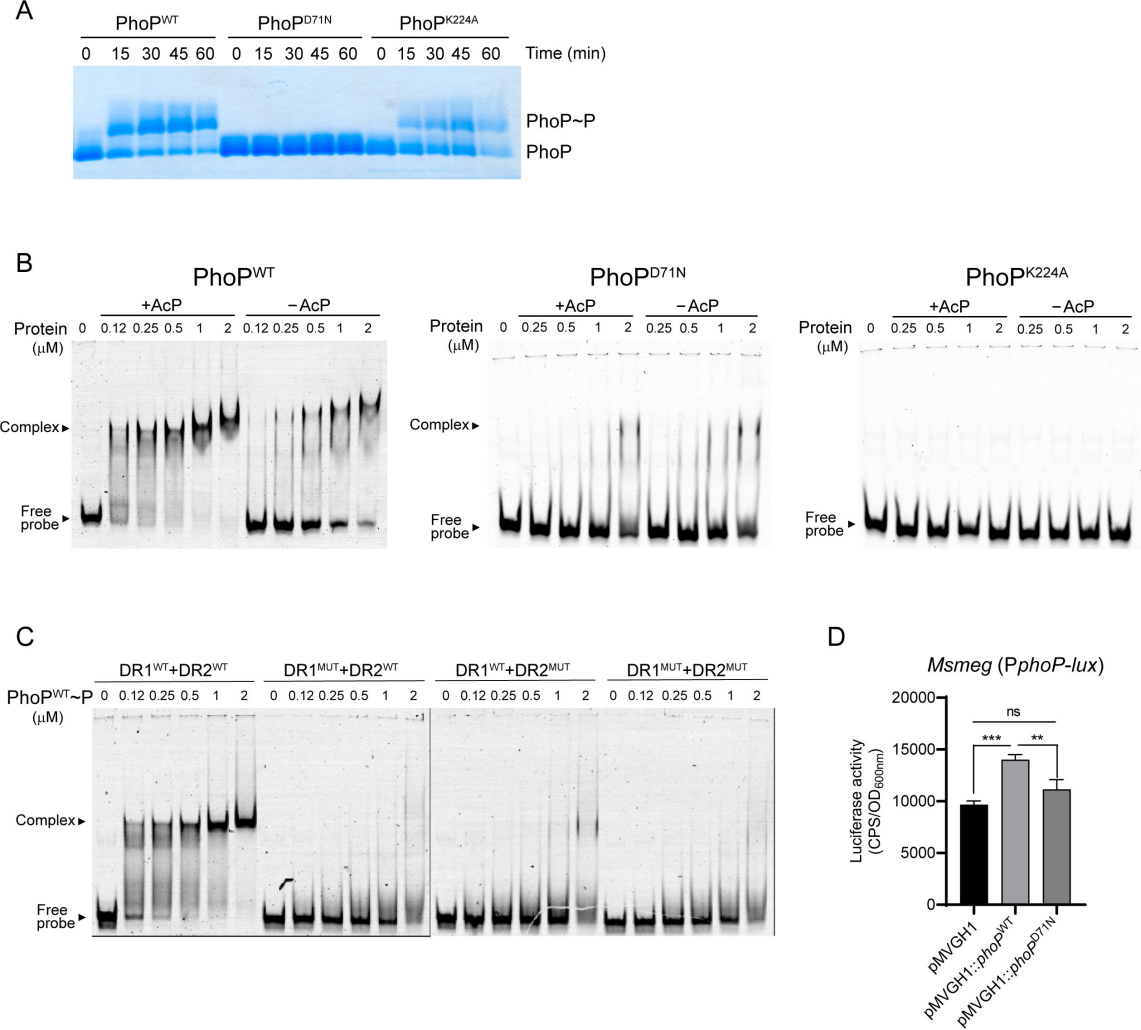
PhoP binding to its own promoter is enhanced by phosphorylation. (**A**) *In vitro* phosphorylation of PhoP^WT^, PhoP^D71N^, and PhoP^K224A^ showing that residue D71 is required for phosphorylation. Recombinant PhoPmas variants were phosphorylated *in vitro* with acetyl-phosphate (AcP) for 0, 15, 30, 45,and 60 min at 37°C. At the indicated time points, samples were mixed with loading buffer and run on a SuperSep Phos-tag 7.5% gel subsequently stained with Coomassie blue. (**B**) EMSA analysis of PhoP^WT^, PhoP^D71N^, and PhoP^K224A^ binding to the promoter region of *phoPmas*. Increasing concentrations of PhoPmas variants, phosphorylated or not with AcP, were incubated with a fluorescently labeled DNA promoter probe (corresponding the WT promoter sequence; see Fig. S4B) for 1 h. Migration of the free and PhoPmas-complexed DNA probe was visualized by in-gel fluorescence. (**C**) The promoter region of *phoPmas* with unmutated DR1 and DR2 regions or the same sequence harboring mutations in either DR1, DR2, or both sites were used as probes. Dilutions of phosphorylated PhoPmas were incubated for 1 h with the fluorescently labeled DNA probes, run for 2 h in a 6% retardation gel and visualized by in-gel fluorescence. (**D**) Luciferase activity was measured in *M. smegmatis* harboring a luciferase reporter gene under control of the *phoPmas* promoter (P*phoP-lux*) and carrying either an empty pMVGH1 replicative plasmid, pMVGH1::*phoP_mas_* allowing for the overexpression of *phoPmas*, or pMVGH1::*phoP_mas_^D71N^* overexpressing a phosphorylation-deficient version of PhoPmas. Cultures were grown to early exponential phase (OD_600nm_ ~ 0.2–0.3) and luciferase activity (counts per second; CPS) was normalized to the OD_600nm_ of the cultures. Results shown are from triplicate cultures. Asterisks denote statistically significant differences between strains pursuant to the unpaired Student’s *t* test (**P < 0.01; ***P < 0.005).

To get basic insights into the DNA-binding properties of PhoP from MABS, electrophoretic mobility shift assays (EMSAs) were conducted using unphosphorylated and phosphorylated purified PhoP protein from *Mmas* (PhoPmas is 100% identical to PhoP from *Mabs* ATCC 19977). Results presented in Fig. 2 show that PhoPmas binds to its own promoter region. Moreover, as reported in *M. tuberculosis* (25 – 28), the DNA-binding activity of PhoPmas was enhanced upon *in vitro* phosphorylation with acetyl phosphate. Mutating the phosphorylation site of PhoPmas, D71 (27), to an asparagine rendered the protein phosphorylation-deficient (Fig. 2A) and dramatically reduced its ability to bind to the promoter region of *phoP* (Fig. 2B). Likewise, mutating the K224 residue of PhoPmas, whose analogous residue in *M. tuberculosis* was shown to be essential for DNA-binding (29) abolished the ability of the response regulator to bind to its promoter under the condition of the assay (Fig. 2B).

PhoP from *M. tuberculosis* (PhoPtb) was shown to regulate its own expression and that of its target genes by binding to direct repeat sequences located in the promoter region of these genes (26, 30 – 32). Given the high level of conservation of PhoPtb and PhoPmas (86% identity and 91% similarity), particularly within their C-terminal DNA-binding region (Fig. S4A), we scanned the *phoP* promoter region from *Mmas* CIP108297 for similar direct repeats and identified two matching motifs (DR1 and DR2) separated by 2 bp (Fig. S4B). The involvement of these sequences in PhoPmas DNA-binding was verified by generating mutated DR1, DR2, or DR1 and DR2 sequences as probes (Fig. S4B) which we used in EMSA assays with phosphorylated PhoPmas. Mutation of either DR1 or DR2 abolished the binding of PhoPmas to its promoter region, supporting the existence of similar binding sites for PhoPtb and PhoPmas (Fig. 2C).

Finally, to determine whether PhoPmas is positively or negatively autoregulated, we resorted to a recombinant *M. smegmatis* strain expressing the promoter region of *phoP* from *Mmas* fused to a luciferase reporter gene (same construct as used in Fig. 1) as a heterologous host for the overexpression of *phoPmas*. Accordingly, the *M. smegmatis* reporter strain was transformed with an empty pMVGH1 replicative plasmid, pMVGH1::*phoPmas* which allows for the constitutive expression of *phoPmas* under control of the strong *hsp60* promoter, or pMVGH1::*phoPmas* ^D71N^ expressing a phosphorylation-deficient version of PhoPmas from the same promoter. Compared to the strain harboring an empty control plasmid, the one overexpressing *phoPmas* showed a 1.5-fold increase in luciferase activity. In contrast, no significant increase in luciferase activity relative to the control strain was observed when phosphorylation-deficient *phoPmas* ^D71N^ was overexpressed (Fig. 2D). Collectively, our results thus suggest that PhoPmas has the ability to upregulate itself and that this activity is enhanced *in vitro* and in intact bacteria by phosphorylation.

### Clinically relevant mutations in PhoR increase the level of expression of *phoP* in a pH-dependent manner

**Fig 3:**
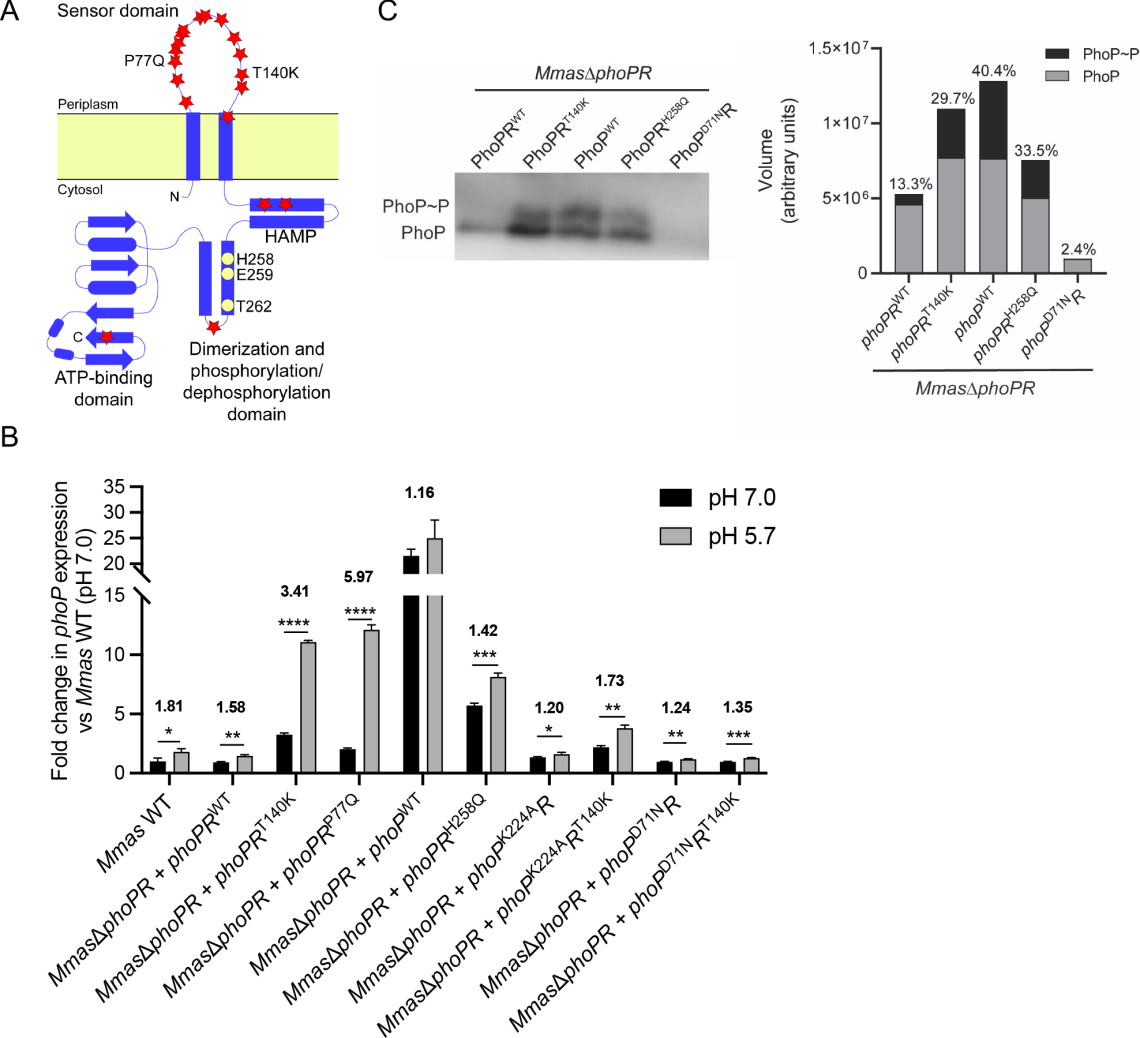
Effect of PhoR on the level of expression of *phoP* at neutral and acidic pH. (**A**) Predicted topology of PhoR from *Mmas* showing the position of the mutations identified in clinical isolates from CF patients (red stars) and the His258/Glu259 and Thr262 residues required for kinase and phosphatase activity, respectively. 70% of the non-synonymous SNPs that accumulated in patients were located in the periplasmic sensor loop. (**B**) The level of expression of *phoP* in WT *Mmas* or *Mmas*Δ*phoPR* complemented with *phoPR* ^WT^, *phoPR* ^T140K^, *phoPR* ^P77Q^, *phoP* ^WT^, *phoPR* ^H258Q^, *phoP* ^K224A^ *R*, *phoP* ^K224A^ *R* ^T140K^ *, phoP* ^D71N^ *R,* or *phoP* ^D71N^ *R* ^T140K^ grown to early exponential phase (OD_600nm_ ~ 0.2–0.3) in minimal medium with 10 mM glucose and 200 µM oleic acid as carbon sources at pH 7.0 (black) or pH 5.7 (gray) was measured by RT-qPCR. Assays were performed in triplicate and are representative of at least two independent experiments. Results are expressed relative to the level of expression of *phoP* in *Mmas* WT grown at pH 7.0 arbitrarily set to 1. Asterisks denote statistically significant differences between culture conditions pursuant to the unpaired Student’s *t* test (*P < 0.05, **P < 0.005, ***P < 0.0005, and ****P < 0.00005). Numbers above the bars indicate the fold change in *phoPmas* expression at acidic pH compared to neutral pH for each strain. (**C**) The amounts of unphosphorylated PhoP (PhoP) and phosphorylated PhoP (PhoP~P) in whole cell lysates from *Mmas*Δ*phoPR* expressing either PhoPR^WT^, PhoPR^T140K^, PhoP^WT^, PhoPR^H258Q^, or PhoP^D71N^R were assessed by running the lysates on Phos-tag gels and revealing the two different forms of PhoP by immunoblot using anti-PhoPmas antibodies. The amounts of PhoP~P and PhoP proteins in each sample were determined by densitometry using AzureSpot and the results are shown on the bar graph. The number above the bars indicates the percentage of PhoP~P in each sample. All cultures were grown in minimal medium with 10 mM glucose and 200 µM oleic acid as carbon sources at pH 5.7 to OD_600nm_ ~ 0.2–0.3.

Mapping of the non-synonymous SNPs accumulated by PhoR during chronic lung infection (A47T, R61W, P77Q, F80L, R83C, T85A, D86E, L105P, P106L, G112C, W127R, S131F, T140K, T141I, D155N, L183P, D209G, Y270H, and D470N) (5) indicated that most of them (>70%) were located in the periplasmic sensor loop of the histidine kinase (HK) with only a few mapping to the catalytic cytosolic domain (Fig. 3A). To probe the requirement of *Mmas* for active forms of PhoP, PhoR, or both components of the TCS in the acidic pH induction of *phoP*, and to analyze the potential effects of patient-derived non-synonymous SNPs on this response, a recombinant approach was used. Accordingly, changes in *phoP* expression at low pH was assessed and compared in *Mmas* recombinant strains generated to recapitulate defects in PhoP DNA-binding, alterations in PhoP or PhoR phosphorylation, complete loss of PhoR, or the expression of patient-derived variants of PhoR (P77Q and T140K). The desired isogenic mutant strains were generated by complementing *Mmas*Δ*phoPR* with either: WT copies of *phoP* and *phoR (phoPR^WT^*); the WT *phoP* gene only (*phoP^WT^*); *phoP^K224A^R* encoding a DNA binding-deficient form of *phoP* (Fig. 2B) and WT form of PhoR; *phoP* ^D71N^ *R* encoding a phosphorylation-deficient form of PhoP and WT form of PhoR (Fig. 2A); *phoPR^H258Q^* encoding a WT form of PhoP and phosphorylation-deficient form of PhoR (30); or *phoPR^P77Q^* and *phoPR^T140K^*, encoding two patient-derived PhoR variants. PhoPR^T140K^ is the same variant as previously used by Bryant et al. (5) to assess the impact of a clinically relevant PhoR mutation on the virulence of *Mmas* in mice. That the different *phoR* gene variants (WT, H258Q, and T140K) were expressed at comparable levels in *Mmas*Δ*phoPR* grown at neutral or acidic pH was verified by RT-qPCR (Fig. S5).

Consistent with the results of the luciferase reporter assay (Fig. 1; Fig. S2), *phoP* expression was ~1.6- to 1.8-fold higher at pH 5.7 than at pH 7.0 in WT *Mmas* and *Mmas*Δ*phoPR* complemented with WT *phoPR* (Fig. 3B). Compared to these two control strains, *Mmas* strains either devoid of PhoR (*Mmas*Δ*phoPR* mutant complemented with *phoP^WT^* only) or expressing a phosphorylation-deficient form of the sensor protein (PhoR^H258Q^) both displayed significantly higher levels of expression of *phoP* (>20-fold) at acidic and neutral pH, with the first strain exhibiting the highest level of expression of the two. However, while the strain expressing PhoR^H258Q^ was still capable of some level of *phoP* induction at pH 5.7 (~1.4-fold induction), the strain devoid of PhoR had essentially lost this ability (~1.1-fold induction) (Fig. 3B). PhoR thus appears to be critical to the control of *phoP* expression and its regulation by pH.

Interestingly, *Mmas* strains expressing the patient-derived variants *phoPR^P77Q^* and *phoPR^T140K^* also expressed *phoP* at significantly higher levels than the isogenic control strain (*Mmas*Δ*phoPR* expressing *phoPR^WT^*) both at pH 5.7 and 7.0. However, compared to other strains (*Mmas*Δ*phoPR* expressing *phoPR^WT^*, *phoPR^H258Q^*, or *phoP^WT^* only)*,* the level of induction of *phoP* at pH 5.7 relative to pH 7.0 was significantly more pronounced with the patient-derived variants (~3.4- to 6-fold) (Fig. 3B). Collectively, these results are suggestive of the reduced ability of PhoR^P77Q^ and PhoR^T140K^ to downregulate *phoP* expression at both neutral and acidic pH, with this effect becoming more pronounced at low pH.

### Proposed model for the regulation of *phoP* expression by PhoR

To explain the results shown in Fig. 3B, we hypothesized that PhoP may efficiently be phosphorylated by other HKs than PhoR (33) or by serine/threonine protein kinases (34, 35) in MABS (as evidenced by the high level of *phoP* expression in the *Mmas* strain devoid of PhoR protein) and that PhoR controls *phoP* expression predominantly by dephosphorylating phospho-PhoP, thereby reducing its ability to autoactivate itself (Fig. 2B). Indeed, similar regulatory models whereby a HK controls the activity of its cognate response regulator by dephosphorylating it has precedents in other bacterial TCS, including the QseBC system of uropathogenic *Escherichia coli* which controls genes involved in virulence (36). In this scenario, the absence of PhoR is expected to lead to maximum expression of *phoP* independent of pH as seen in Fig. 3B, while mutating the phosphorylation residue of PhoR (H258) should reduce (but not completely abolish) its phosphatase activity at all pHs (37, 38) resulting in enhanced *phoP* expression (as seen in Fig. 3B in *Mmas*Δ*phoPR* expressing *phoPR^H258Q^*). In keeping with this model, PhoR^P77Q^ and PhoR^T140K^, similar to PhoR^H258Q^, appear to be less capable of dephosphorylating phospho-PhoP at both pHs but this deficiency is exacerbated at low pH, resulting in significantly higher levels of expression of *phoP* at pH 5.7 relative to pH 7.0 (3.4- to 6-fold compared to 1.4-fold induction in the strain expressing *phoPR^H258Q^*, and 1.6- to 1.8-fold induction in strains expressing WT forms of PhoR) (Fig. 3B). Consistent with the phosphorylation state of PhoP being at the core of its pH-dependent autoregulation, mutating the phosphorylation site of PhoP (as in the strain expressing *phoP^D71N^R^T140K^*) abolished both the higher level of expression of *phoP* in the PhoR^T140K^ mutant and the ability of this mutant to upregulate *phoP* at acidic pH more than 1.35-fold (Fig. 3B). Mutating a residue important for the ability of PhoP to bind DNA (as in the strain expressing *phoP^K224A^R^T140K^*) similarly reduced *phoP* expression at pH 7.0, yet, did not abolish the ability of the mutant to upregulate *phoP* at low pH (Fig. 3B). We tentatively attribute this result to the fact that the increased phosphorylation of PhoP in this strain enhances the dimerization of PhoP and, thus, its ability to bind DNA (26), even in the background of a mutated K224 residue.

With the goal to provide more direct experimental support for our model, we first assessed the phosphorylation state of PhoP in *Mmas*Δ*phoPR* expressing either *phoPR^WT^*, *phoP^WT^* (without *phoR*), *phoP* ^D71N^ *R*, *phoPR^H258Q^*, or *phoPR^T140K^* grown at acidic pH using Phos-tag gels which can resolve phosphorylated and unphosphorylated forms of PhoP in bacterial cell lysates. In line with the higher level of expression of *phoP* in the strains expressing *phoP^WT^*, *phoPR^H258Q^*, and *phoPR^T140K^* relative to *Mmas*Δ*phoPR* expressing *phoPR^WT^* (Fig. 3B), more PhoP protein was detected in the first three strains (Fig. 3C). Furthermore, relative to the control strain expressing *phoPR^WT^* in which only ~13% of PhoP was phosphorylated, more than ~30% of the PhoP protein detected in lysates prepared from *Mmas*Δ*phoPR* expressing *phoP^WT^*, *phoPR^H258Q^*, and *phoPR^T140K^* was phosphorylated (Fig. 3C). Thus, the level of expression of *phoP* (and total amount of PhoP protein produced) positively correlated with its degree of phosphorylation (Fig. 3B–C). The ratio of phospho-PhoP to PhoP was the highest (~40%) in the strain totally deficient in *phoR* suggesting that PhoR is required to maintain PhoP in an unphosphorylated, and thus inactive, state in *Mmas*.

**Fig 4:**
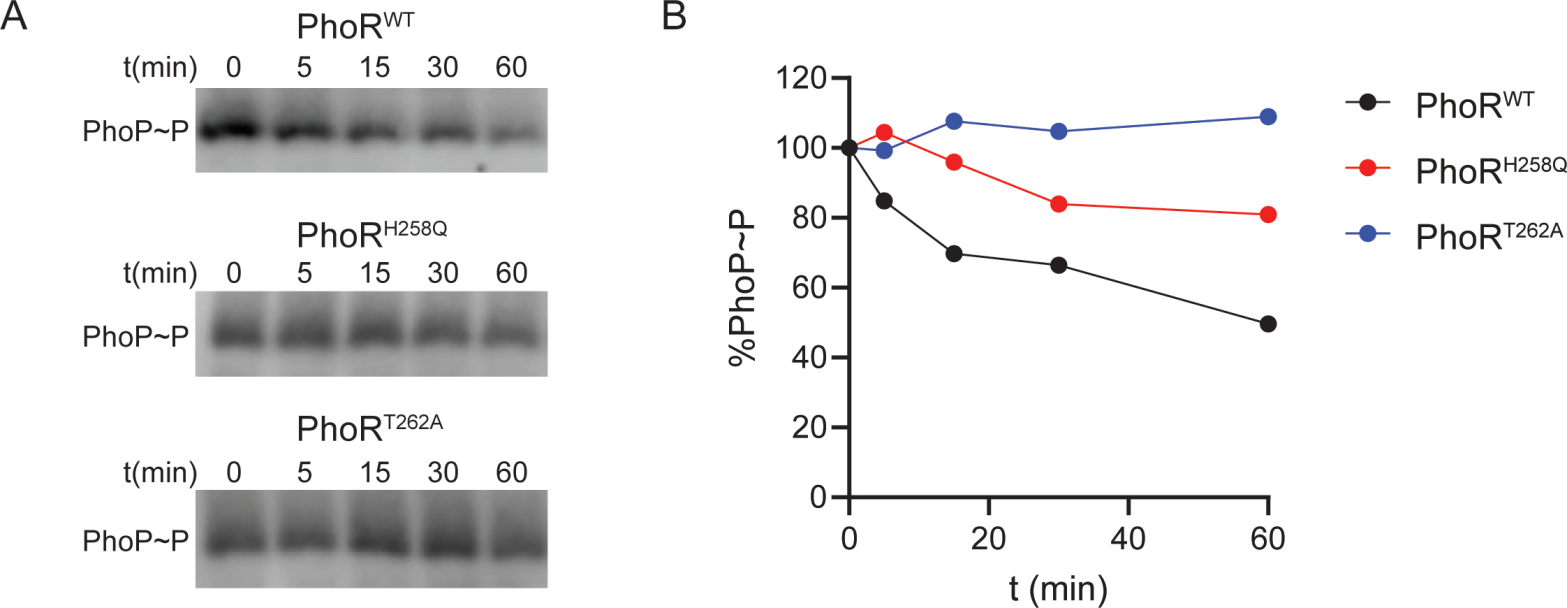
Phosphatase activity of PhoR. (**A**) The phosphatase activity of purified PhoR^WT^, PhoR^H258Q^, and PhoR^T262A^ was measured by incubating the purified cytosolic catalytic domain of the enzymes with PhoP~P for the indicated times. Samples were run on Phos-tag gels and analyzed by western blot with anti-PhoP antibodies. (**B**) Quantification of PhoP~P shows PhoR^H258Q^ has reduced activity compared to PhoR^WT^, while the activity of PhoR^T262A^ is completely abolished. The results presented are representative of two independent experiments using different enzyme preparations.

The ability of PhoR to dephosphorylate phospho-PhoP *in vitro* was next directly tested by expressing and purifying the cytoplasmic C-terminal catalytic domain of PhoR in *E. coli* (Fig. 3A) and using it in a phosphatase assay where phospho-PhoP served as the substrate. The results which are shown in Fig. 4 confirmed that PhoR dephosphorylates phospho-PhoP in a time-dependent manner. This activity was lost when the essential phosphatase residue of PhoR, T262 was mutated to an alanine and decreased when the kinase residue of PhoR, H258 was mutated to a glutamine.

Collectively, our result supports a regulatory model wherein PhoR from MABS controls PhoP expression through its dephosphorylation in a pH-dependent manner.

### Effect of clinically relevant PhoR mutations on the gene expression profile of *Mmas*

In order to delineate the PhoPR regulon of *Mmas* and determine how clinically relevant mutations in PhoR may alter response to pH, RNA sequencing was used to compare the gene expression profiles of *Mmas* WT, *Mmas*Δ*phoPR*, and isogenic strains expressing either PhoPR^WT^, PhoPR^H258Q^, PhoPR^T140K^, or PhoP^WT^, at both neutral (pH 7.0) and acidic (pH 5.7) pH.

Analysis of differentially expressed (DE) genes in *Mmas*Δ*phoPR* relative to *Mmas* WT [Log_2_ fold change (FC) ≥1 or ≤1 with *P*-adj < 0.05] revealed 40 downregulated genes and 20 upregulated genes at acidic pH, and 22 downregulated genes and 22 upregulated genes at neutral pH (Table 1; Table S1). Seven upregulated genes and 15 downregulated genes (highlighted in Table 1 and Table S1) were differentially expressed at both pHs. Strikingly, a significant number of DE genes of known or predictable function were related to redox stress. Notable among the downregulated genes at both pHs in *Mmas*Δ*phoPR* is the transcriptional regulator gene *whiB3* (*MAB_3726*) whose ortholog in *M. tuberculosis* is regulated by PhoP and encodes a cytosolic redox sensor required for survival under acidic conditions (39, 40). WhiB3 from *M. tuberculosis* controls a number of genes involved in modulating phagosome maturation and the maintenance of redox homeostasis in response to acidic pH (39).

**TABLE 1:** Genes found to be differentially expressed in *Mmas*Δ*phoPR* versus WT *Mmas* at pH 5.7

Gene number	Gene product	log_2_ FC	*Mtb* H37Rv ortholog
**Upregulated genes**			
***MAB_0049***	**EsxH**	**1.11**	*Rv0288 (esxH*)
*MAB_0354*c	Oxidoreductase, 2-nitropropane dioxygenase family	1.50	*Rv1894c*
***MAB_0650***	**60 kDa chaperonin 2 (Protein Cpn60 2) (GroEL)**	**1.20**	*Rv0440* (*groEL2*)
***MAB_0665***	**EsxG**	**1.39**	*Rv0287 (esxG*)
*MAB_0885*c	19 kDa lipoprotein lpqH precursor	1.01	*Rv3763* (*lpqH*)
***MAB_1013*** *	**Hypothetical protein**	**1.19**	–
MAB_1190*	Hypothetical protein	1.22	*Rv1072*
*MAB_1196*	Proline-rich antigen (36 kDa antigen)	1.11	*Rv1078*
*MAB_1241*c	Hypothetical protein	1.25	–^*b*^
*MAB_1287*	Probable conserved membrane protein, MmpL family	1.17	–
*MAB_2629*	Hypothetical protein	1.13	*Rv0310c*
*MAB_3219*	Hypothetical protein	1.13	–
***MAB_3467*** c*	**18 kDa antigen (HSP 16.7)**	**1.67**	–
***MAB_3732*** **c**	**10 kDa chaperonin (GroES)**	**1.55**	*Rv3418c*
***MAB_3946*** **c**	**Putative short chain dehydrogenase/reductase**	**1.16**	*Rv3224*
*MAB_4284*c	Hypothetical collagen-like protein	1.25	–
*MAB_4429*	Hypothetical protein	1.68	–
*MAB_4446*	Hypothetical protein	1.13	*Rv3773c*
*MAB_4555*c	Possible glyoxalase/bleomycin resistance protein/dioxygenase	1.14	–
*MAB_4770*c	Hypothetical protein	1.05	–
**Downregulated genes**			
*MAB_0018*c	Putative methyltransferase	−2.52	–
***MAB_0513***	**Putative monooxygenase**	**−2.79**	–
***MAB_0514***	**Probable oxidoreductase EphD**	**2.44**	–
***MAB_0673*** *	**DNA-binding response regulator PhoP**	**−13.97**	*Rv0757* (*phoP*)
***MAB_0674***	**Sensor histidine kinase PhoR**	**−5.53**	*Rv0758* (*phoR*)
*MAB_0694*	Hypothetical protein	−1.27	*Rv0784*
***MAB_1587*** **c**	**Probable fatty acid desaturase**	**−2.39**	–
***MAB_1588*** **c**	**Putative oxidoreductase**	**−1.64**	–
***MAB_1589***	**Putative transcriptional regulator, TetR family**	**−1.86**	–
*MAB_1591*	Hypothetical protein	−1.03	*Rv1118c*
*MAB_1789*	Bacteriophage protein	−1.55	–
*MAB_1793*	Phage associated (putative structural protein)	−1.99	–
***MAB_2277***	**Probable aldehyde dehydrogenase**	**−1.31**	–
***MAB_2509*** **c**	**Hypothetical protein**	**−1.47**	–
*MAB_2551*	Hypothetical protein	−1.12	–
MAB_2590*	Putative polysaccharide deacetylase	−1.03	–
*MAB_3358*c	1-acyl-sn-glycerol-3-phosphate acyltransferase	−1.64	*Rv3026c*
***MAB_3726*** *	**Putative transcriptional regulator, WhiB3**	**−3.06**	*Rv3416* (*whiB3*)
***MAB_3918*** **c**	**Putative oxidoreductase**	**−1.88**	–
***MAB_3919*** **c**	**Putative short chain dehydrogenase/reductase**	**−2.00**	–
***MAB_3920*** **c**	**Probable monooxygenase**	**−2.35**	*Rv3049c*
***MAB_3921*** **c**	**Diiron oxygenase**	**−1.94**	–
***MAB_3922*** **c**	**Diiron oxygenase**	**−2.63**	–
*MAB_3923*	Hypothetical protein	−2.15	–
*MAB_4071*	Hypothetical protein	−1.01	–
*MAB_4303*	Glycoside hydrolase family 27 protein	−1.03	–
*MAB_4399*c*	Hypothetical protein	−1.65	–
*MAB_r5053*	5S ribosomal RNA	−1.02	–
*MAB_t5003*	tRNA-Leu(CAG)	−1.26	–
*MAB_t5004*	tRNA-Ser(TGA)	−1.63	–
*MAB_t5007*	tRNA-Ser(CGA)	−1.51	–
*MAB_t5013*	tRNA-Glu(TTC)	−1.24	–
*MAB_t5014*	tRNA-Asp(GTC)	−1.15	–
*MAB_t5020c*	tRNA-Arg(CCG)	−1.69	–
*MAB_t5021*	tRNA-Leu(TAG)	−1.24	–
*MAB_t5031c*	tRNA-Leu(GAG)	−1.37	–
*MAB_t5035c*	tRNA-Val(CAC)	−1.20	–
*MAB_t5037*	tRNA-Cys(GCA)	−1.18	–
*MAB_t5038*	tRNA-Val(GAC)	−1.30	–
*MAB_t5039c*	tRNA-Glu(CTC)	−1.13	–

^*a*^ Differentially expressed genes in *Mmas*Δ*phoPR* compared to *Mmas* WT at pH 5.7 (Log_2_ Fold Change (LFC) ≥1 or ≤−1, *P*adj < 0.05). Genes that were also differentially expressed at pH 7.0 are in bold. Asterisks denote genes whose promoters harbor a putative PhoP binding site (DR1 + DR2) (see Fig. S4B).

^*b*^ –, no ortholog gene in *M. tb*.

Mycobacteria residing within the hostile environment of acidified phagosomes are indeed known to be exposed to reductive stress which, in other prokaryotes, has been reported to have deleterious effects on protein synthesis and folding (41). Consistent with the idea that the *phoPR* KO mutant is impaired in its ability to maintain redox homeostasis at acidic pH, three chaperonins (*MAB_0650*, *MAB_3467*c, and *MAB_3732*c) were induced in the mutant at pH 5.7, suggestive of protein aggregation or misfolding. Concurrently, a number of tRNAs were downregulated suggestive of altered protein synthesis, as were three genes potentially involved in fatty acid and polysaccharide biosynthesis (*MAB_1587*c, *MAB_2590*, and *MAB_3358*c).

The PhoPR TCS of *M. tuberculosis* has been implicated in the regulation of different protein export pathways, including the ESX-1 type-VII secretion system which controls the secretion of ESAT-6, a major virulence factor that interferes with phagosomal maturation (21). Interestingly, two genes (*MAB_0049* encoding an ESAT-6-like protein and *MAB_0665* encoding a PE family protein) with similarities to type-VII secretion system proteins were upregulated in the *Mmas phoPR* KO at both pHs. While the precise functions of MAB_0049 and MAB_0665 are not known, these two proteins closely resemble the ESX-3 EsxH and EsxG proteins from *M. tuberculosis* which have been involved in iron acquisition and virulence (42, 43). What is more, three 99%–100% identical copies of the *esxH* and *esxG* genes are present in the *Mabs* ATCC 19977 and *Mmas* CIP108297 genomes, with one set located in the ESX-3 gene cluster (genes *MAB_2224-MAB_2234*c in *Mabs* ATCC 19977).

**Fig 5:**
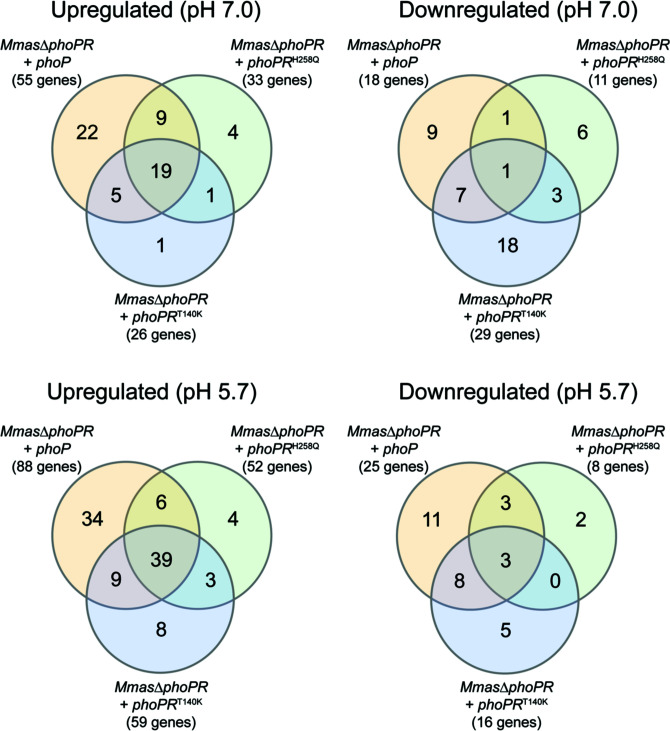
The transcriptional profile of *Mmas* expressing a patient-derived PhoR mutant shows a significant overlap with that of strains totally or partially deficient in PhoR activity. Venn diagram showing the number of genes upregulated (Log_2_ Fold Change (LFC) ≥1, left) or downregulated (LFC ≤ −1, right) in *Mmas*Δ*phoPR* expressing *phoP^WT^*, *phoPR* ^H258Q^ or *phoPR* ^T140K^ relative to *Mmas*Δ*phoPR* expressing *phoPR* ^WT^. Cells were grown in minimal medium with 10 mM glucose and 200 µM oleic acid as carbon sources at pH 7.0 (top) or pH 5.7 (bottom).

**Fig 6:**
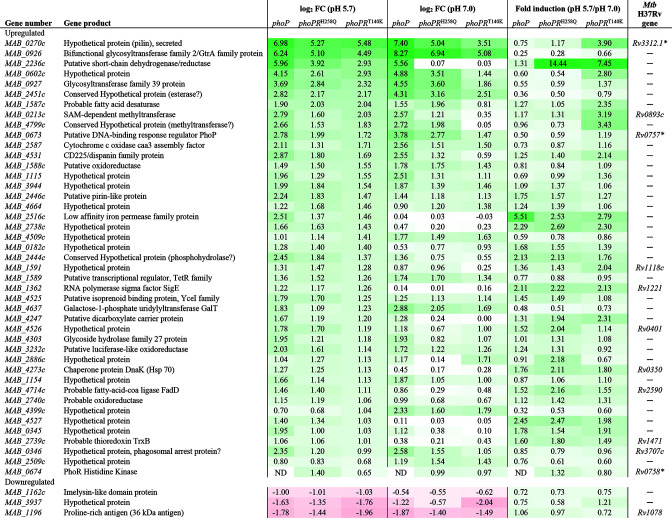
Differentially expressed genes in strains partially or totally deficient in PhoR activity relative to *Mmas* expressing WT *phoR*. Only the genes found to be significantly up- or down-regulated (Log_2_ Fold Change (LFC) ≥ 1 or ≤ −1, *P*adj <0.05) in all three *phoR* mutant strains (i.e., *Mmas*Δ*phoPR* expressing *phoP* ^WT^, *phoPR* ^H258Q^ or *phoPR* ^T140K^) compared to *Mmas*Δ*phoPR* expressing *phoPR* ^WT^ at pH 5.7 or pH 7.0 were included in the figure. The right column shows the fold change in the expression of each gene at pH 5.7 compared to pH 7.0 for each strain. Asterisks denote genes that are also under control of PhoP in *M. tuberculosis*.

To determine how clinically relevant mutations in PhoR impacted the gene expression profile of *Mmas* at acidic and neutral pH, we next compared the transcriptional profiles of *Mmas*Δ*phoPR* expressing *phoPR* ^T140K^ to that of *Mmas*Δ*phoPR* expressing *phoPR* ^WT^ at both pHs. *Mmas*Δ*phoPR* expressing *phoP* ^WT^ only and *Mmas*Δ*phoPR* expressing *phoPR* ^H258Q^ were also included in these analyses as two comparator strains, respectively, totally and partially deficient in PhoR activity. As shown in Fig. 5, all three mutant strains showed a significant DE gene overlap relative to *Mmas*Δ*phoPR* expressing *phoPR* ^WT^ at both pHs, particularly in the upregulated genes category. Upregulated and downregulated genes in each strain at both pHs are listed in Table S2. Figure 6 shows the list of DE genes found to be upregulated or downregulated in all three mutant strains relative to *Mmas*Δ*phoPR* expressing *phoPR* ^WT^ at pH 5.7 and 7.0. Consistent with the qPCR results presented in Fig. 3, *phoP* was among the upregulated genes in all three mutant strains at both pHs. The last three columns of Fig. 6 (ratio of Log_2_ FC in gene expression at pH 5.7 vs 7.0) further show that the majority of the upregulated genes were more strongly induced when pH dropped to 5.7 in the strains presenting mutations in PhoR than in *Mmas* expressing PhoPR^WT^. This was particularly true in the strain expressing PhoPR^T140K^ with 32 out of 42 genes more strongly induced. Seven upregulated genes (excluding *phoP*) were the same ones whose expression was reduced in *Mmas*Δ*phoPR* compared to WT *Mmas* (Table 1) and included the putative fatty acid desaturase-related gene cluster (*MAB_1587c-MAB_1591*). The upregulation of two glycosyltransferase genes (*MAB_0926* and *MAB_0927*), a fatty acid-CoA ligase gene (*MAB_4714*c), and a gene involved in galactose metabolism were further indication of likely changes in the fatty acid and polysaccharide metabolism in the *phoR* mutants. Other upregulated genes clearly signaled redox stress and included a chaperonin (*MAB_4273*c), a cytochrome c oxidase assembly factor (*MAB_2587*), a probable thioredoxin gene (*MAB_2739*c), and the sigma factor *sigE* (*MAB_1362*) whose ortholog in *M. tuberculosis* interacts with PhoP to control acid-induced redox homeostasis (44). Finally, three upregulated genes stood out in Fig. 6 for their potential to promote the intracellular survival of *Mmas* PhoR mutants. The first one, *MAB_0270*c, is thought to participate in the formation of pili-like structures and its ortholog in *M. tuberculosis* (Mpt; Rv3312A) was found to be under the control of PhoP (11, 45). Mpt binds to laminin and was shown to play a role in the ability of *M. tuberculosis* to adhere to and invade epithelial cells (46, 47). The second one, *MAB_0346*, encodes an *in vivo*-induced exported protein whose orthologs in *M. bovis* BCG and *M. tuberculosis* (*Rv3707c*) were implicated in the arrest of phagosomal acidification in macrophages and intracellular growth (48, 49). The third gene, *MAB_4531*, has no ortholog in *M. tuberculosis* and encodes an interferon-induced transmembrane-like protein whose functional homologs in vertebrates confer protection not only against certain viral infections but also *M. tuberculosis* infection by enhancing endosomal acidification in infected cells (50, 51). It is tempting to speculate that MAB_4531 may be another MABS protein capable of interfering with phagosomal acidification. RT-qPCR confirmed the RNA-seq data for five DE genes, including *phoP*, *phoR*, *MAB_0926*, *MAB_1115*, and *MAB_4531* (Fig. S5).

**Fig 7:**
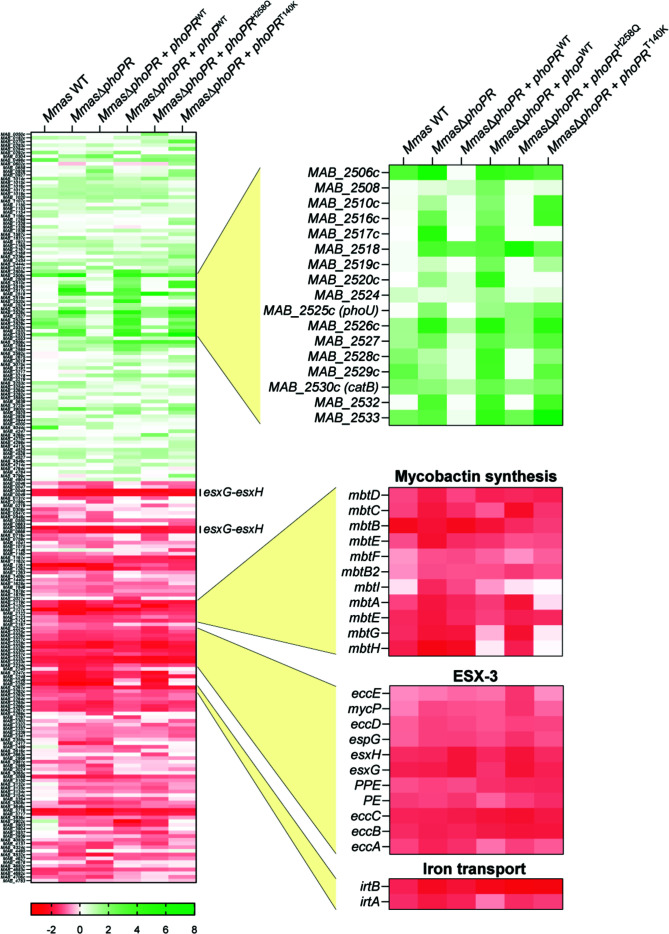
Comparison of the acidic pH transcriptional response of WT *Mmas*, the *phoPR* knock-out mutant and *Mmas*Δ*phoPR* expressing different mutated forms of *phoR*. Heatmap representation of the general response to acidic pH of *Mmas* WT, *Mmas*Δ*phoPR*, and *Mmas*Δ*phoPR* complemented with *phoPR* ^WT^, *phoP* ^WT^, *phoPR* ^H258Q^, or *phoPR* ^T140K^. Every gene with an LFC ≥1 or ≤−1 (*P*adj < 0.05) in at least one strain is represented on the heatmap. Details of the heatmap are shown for a few selected genes or pathways found to be enriched in our analyses.

Comparing more generally the ability of all *Mmas* strains analyzed in this report (*Mmas* WT, *Mmas*Δ*phoPR*, and *Mmas*Δ*phoPR* complemented with *phoPR* ^WT^ and *Mmas*Δ*phoPR* complemented with the various mutated forms of *phoP* or *phoR*) to respond to acidic pH, a striking observation from the heatmap presented in Fig. 7 was the fact that, overall, all of them (including the *phoPR* KO) underwent similar changes in their transcriptional profiles at pH 5.7 versus pH 7.0 (Fig. 7 and Table S3). While we do not exclude that performing the same experiment within the first few minutes following the exposure of *Mmas* strains to acidic pH would not reveal greater differences between strains, these results indicate that all *Mmas* strains are eventually capable of adjusting to acidic pH by up or -down-regulating a similar set of genes. The expression of these genes is thus likely to be under the control of both PhoPR-dependent and PhoPR-independent regulatory mechanisms. In line with their comparable response to acidic pH, all strains grew similarly to the controls expressing WT forms of *phoPR* in medium adjusted to pH 5.7 (Fig. S6). Notable among the sets of genes downregulated at acidic pH were mycobactin biosynthetic genes, two iron importer genes (*irtA* and *irtB*) and ESX-3 genes (including all three sets of *esxH*/*esxG* genes) required for mycobactin-mediated iron acquisition (42) (Fig. 7). Low pH increases the solubility of transition metals like iron, thereby allowing this metal to cross biological membranes and participate in metal-catalyzed ROS generation via the Fenton reaction. The observed downregulation of iron acquisition systems in *Mmas* under acidic pH thus most likely reflects an effort to mitigate ROS stress.

### Impact of clinically relevant PhoR mutations on MABS uptake by macrophages and intracellular replication

**Fig 8:**
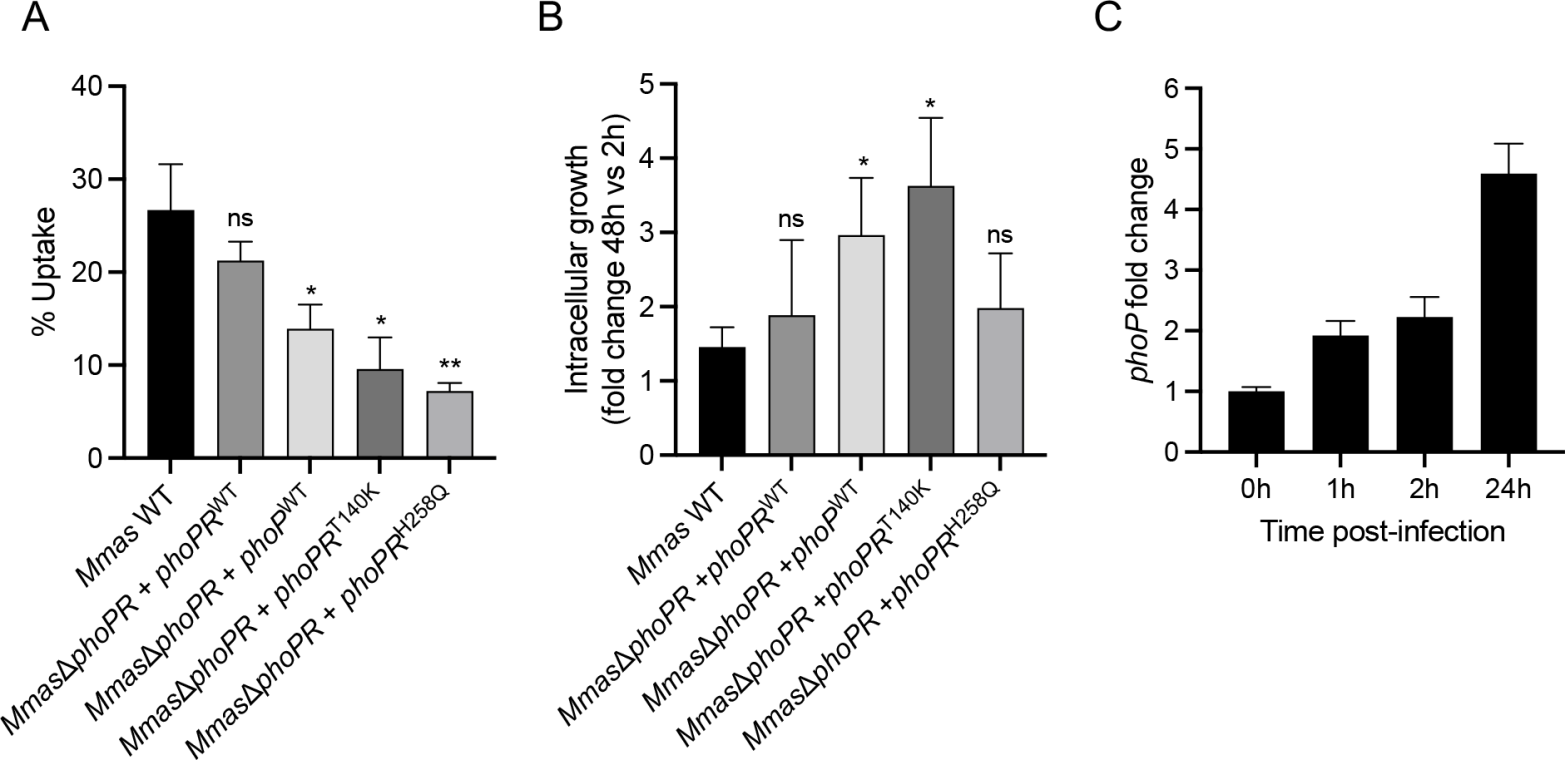
Effects of PhoR mutations on the uptake of *Mmas* by macrophages and intracellular replication. WT *Mmas* and *Mmas*Δ*phoPR* complemented with *phoPR* ^WT^, *phoP* ^WT^, *phoPR* ^T140K^, or *phoPR* ^H258Q^ were incubated with human THP-1 monocyte-derived macrophages at an MOI of 1. Two and 48 h post-infection, the macrophages were lysed and intracellular bacteria enumerated by CFU plating. (**A**) shows the percentage of bacteria in the inoculum that were internalized after 2 h. (**B**) shows the fold change in CFUs for each strain between 2 and 48 h. The results presented are the means (±SD) of triplicate wells from one experiment and are representative of two independent experiments. Asterisks denote statistically significant differences between *Mmas* WT and recombinant strains pursuant to the unpaired Student’s *t* test (*P < 0.05; **P < 0.005; ns, not-significant). (**C**) *phoP* expression following infection of THP-1 cells with WT *Mmas* CIP108297 was monitored by RT-qPCR over time. THP-1 cells were infected for 2 h, followed by 1 h treatment with amikacin to eliminate extracellular bacteria. After this treatment (defined as time 0), cells were collected at different time points. Results are expressed as fold changes over the level of expression of *phoP* measured at time 0. The results were standardized to *sigA* expression levels and are shown as means ± standard deviations from biological triplicates (*n* = 3 RNA extractions and RT-qPCR reactions).

Given the overlap in the transcriptional profiles of the strains displaying alterations or loss of PhoR catalytic function (strain expressing *phoP^WT^* only, *phoR* ^T140K^ and *phoR* ^H258Q^), we next sought to compare these strains for internalization and survival inside human THP-1 monocyte-derived macrophages. The results show that expression of PhoR^T140K^ or PhoR^H258Q^ mimic the absence of PhoR, all three mutations significantly decreasing the uptake of *Mmas* by macrophages (Fig. 8A) while increasing intracellular survival (Fig. 8B). The finding shows that the strain harboring a sensor loop mutation in PhoR (PhoR^T140K^) exhibited similar phenotypes with regards to macrophage invasion and intracellular replication as *Mmas* lacking PhoR completely, reinforces our prevailing hypothesis that clinically relevant mutations in PhoR cause a dramatic decrease in the phosphatase activity of this protein under host-relevant conditions.

In line with the idea that genes positively regulated by PhoP promote the intracellular survival of *Mmas*, and in contrast to previous observations made in *Mabs*-infected J774.2 macrophages (24), a clear upregulation of *phoP* was observed within the first 24 h following THP-1 infection by WT *Mmas* (Fig. 8C).

## DISCUSSION

**Fig 9:**
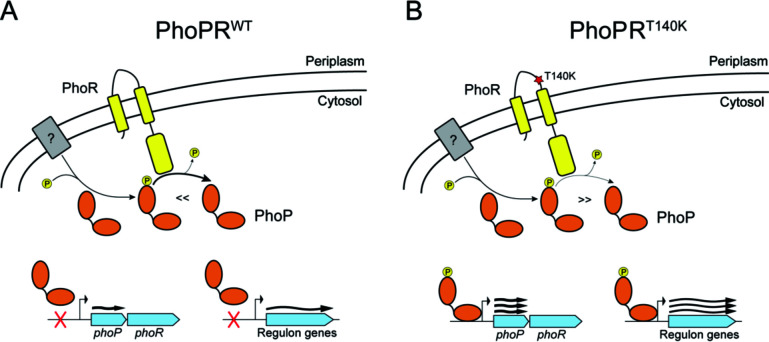
Proposed model depicting the regulation of PhoP by PhoR in *Mmas* and the effects of patient-derived mutations in PhoR. (**A**) In the absence of a stimulus, PhoR primarily acts as a phosphatase driving the dephosphorylation of phospho-PhoP (which may be phosphorylated by other as yet unknown HKs or serine/threonine protein kinases). Dephosphorylated PhoP has low affinity for DNA resulting in the limited expression of *phoP* and PhoP-regulated genes. (**B**) Clinically relevant mutations in PhoR decrease its phosphatase activity, particularly at acidic pH, causing phospho-PhoP to accumulate in the cells and *phoPR* and regulon to be induced.

The finding that a small set of MABS genes is under strong evolutionary pressure during human infection (5) has provided unique opportunities to unravel the critical molecular determinants that have allowed MABS to emerge as a chronic pathogen of the lung. The results of our study support an important role for the TCS PhoPR in host-adaptation and provide insights into the underlying mechanisms. We find that the response regulator PhoP is induced in response to acidic pH, a stress encountered by MABS inside host phagocytic cells and in the acidified airway of patients with CF, in a PhoP-dependent and PhoR-dependent manner. Moreover, our findings support a model wherein the dephosphorylation of phospho-PhoP by its cognate HK, PhoR, leads to reduced *phoP* expression which is partially reverted at acidic pH when the phosphatase activity of PhoR decreases (Fig. 9). The derepression of *phoP* at low pH leads to the upregulation of a number of genes, many of which appear to play a role in redox homeostasis or to more directly enhance intracellular survival by modulating phagosomal maturation. Consistent with this observation, *Mmas* strains that constitutively or inducibly express *phoP* at the highest levels at pH 5.7 tend to be more virulent and to be less efficiently taken up by THP-1 cells. Non-synonymous SNPs identified in the *phoR* gene of host-adapted isolates cause *phoP* and associated genes to be more strongly upregulated at low pH, suggesting that the phosphatase activity of PhoR in these isolates is probably more susceptible to inactivation by acidic pH than that of strains expressing WT PhoR. One of the outcomes of this phenotype highlighted by our macrophage infection studies is the greater ability of isolates expressing mutated PhoR variants to evade macrophage killing.

The fact that MABS isolates harboring mutations in PhoR were found at a significantly higher rate in patients with pulmonary MABS disease than in patients with MABS wound infections suggest that alterations in the activity of PhoR may be particularly advantageous in the lung environment (5). The marked increase in virulence of the *Mmas* strain expressing *phoPR^T140K^* [annotated as PhoPRΔ::PhoPRmut in reference (5)]. compared to *Mmas* expressing WT *phoPR* in mouse lungs lends support to this hypothesis (5). A correlate of this is that the activation of PhoPR may be governed by other signals besides acidic pH, some of which may be specific to the lung environment and, possibly, not fully recapitulated in our macrophage model.

Such a TCS-based control of bacterial pathogenesis is not unprecedented. As mentioned earlier, QseC from uropathogenic *E. coli* modulates the expression of virulence genes by dephosphorylating its cognate response regulator, QseB (36). Like QseC, PhoRmas belongs to the HisKA subfamily of HKs that have been reported to display both kinase and phosphatase activity on their cognate transcriptional regulator (38, 52). In line with this classification, the cytosolic domain of PhoRmas harbors an HD/E-X-X-T/N motif where the phosphorylatable histidine residue (H258 in PhoRmas) and adjacent glutamic acid residue (E259) are required for kinase activity and the asparagine or threonine residue (T262 in PhoRmas) is thought to be essential for phosphatase activity (52) (Fig. 3A). While not essential to the phosphatase function of the HK, the histidine residue of the consensus catalytic motif has been shown to enhance this activity (37, 38). Our *in vitro* enzymatic assays with PhoRmas were able to confirm both the essentiality of T262 for phosphatase activity of the protein and the requirement for H258 for optimal activity (Fig. 4). The negative effect of acidic pH on the phosphatase activity of HisKA family HKs was attributed to the ability of the histidine residue to act as a general base or as a nucleophile in the reactions catalyzed by the HK (37, 38). As indicated above, our results suggest that the patient-derived non-synonymous SNPs found in MABS PhoR cause the phosphatase activity of the protein to be reduced even more dramatically at low pH. We speculate that this effect may either result from pH-induced conformational changes in PhoRmas (at least in the case of mutations in the extracellular sensor domain) or from alterations more directly impacting the HK catalytic site (in the case of mutations located in the vicinity of the conserved cytoplasmic catalytic domain; e.g., Y270H) (Fig. 3A).

The fact that the sensor loop of PhoR also accumulates a higher than expected number of non-synonymous consensus SNPs in clinical isolates of *M. tuberculosis* (10) suggests that fine-tuning the activity of PhoR in response to host-relevant stresses may be a conserved *Mycobacterium* mechanism of adaptation to the lung environment. Both the PhoPR TCS of *M. tuberculosis* and that of MABS are induced at acidic pH (14, 33) and contribute to counteracting pH-associated redox stress. As in MABS, phospho-PhoP from *M. tuberculosis* activates the transcription of a number of genes, including those involved in the biosynthesis of TB complex-specific lipids known as sulfolipids, di- and poly-acyltrehaloses (21). Based on the fact that *phoP* but not *phoR* is required for the synthesis of these lipids, we proposed earlier that *M. tuberculosis* PhoR may control the expression of PhoP and regulon by dephosphorylating phospho-PhoP rather than phosphorylating PhoP (13). This hypothesis recently gained further support as PhoRtb was shown to catalyze *in vitro* the dephosphorylation of phospho-PhoP (33). It is thus tempting to speculate that the non-synonymous SNPs accumulated by PhoR from *M. tuberculosis* also serve to modulate the phosphatase activity of this protein. Owing to the number of differences that exist between the PhoPR TCS of *M. tuberculosis* and MABS, however, this assumption will require careful validation in dedicated isogenic *M. tuberculosis* mutants. One important difference between *M. tuberculosis* and MABS is the fact that the disruption of *phoPR* in MABS enhances virulence (5) rather than attenuates it (11 – 14). We do not have any obvious explanation for this phenotype at this point. Among other hypotheses, it is possible that differences in the gene expression profile of the *phoPR* KO compared to WT *Mmas* that may not have been revealed under the particular stress condition used in our RNAseq studies contribute to this phenotype. Another difference between the two mycobacterial species resides in the fact that PhoPR was found to be required to slow the growth of *M. tuberculosis* at acidic pH in the presence of certain carbon sources (16), whereas we observed no such phenotype in MABS. Unlike the situation in *M. tuberculosis*, the growth of our *Mmas*Δ*phoPR* mutant *in vitro* was also not slowed when magnesium concentration dropped below 0.16 mM (11). A comparison of the lists of genes under (direct or indirect) control of PhoPR and the hypoxia-controlled two-component regulator DosRS in MABS (53, 54) further failed to reveal any overlap between the two lists, suggestive of an absence of crosstalk between the two TCS, unlike the situation in *M. tuberculosis* (17, 18). Finally, whereas PhoPR from *M. tuberculosis* regulates, directly or indirectly, the synthesis of a number of surface and intracellular lipids associated with virulence and persistence (11, 13, 14), we failed to observe any notable changes in the lipid profile of *Mmas* upon *phoPR* disruption, whether the strains were grown at neutral or acidic pH (Fig. S7). In line with this result, the *phoPR* mutant of *Mmas* displayed a drug susceptibility profile comparable to that of the WT parent strain (Table S4) indicating that the permeability of its cell envelope was not significantly altered. Importantly, the fact that *Mmas* strains expressing patient-derived forms of *phoPR* (PhoPR^T140K^ and PhoPR^P77Q^) displayed WT susceptibility to a number of clinically used antibiotics further indicates that drug resistance was not the primary driver of the selection of non-synonymous SNPs in PhoR during infection.

While it is clear from the results presented herein that the PhoPR TCS of MABS has evolved to become an important player in the ability of the bacterium to evade immunity and survive during host infection, much remains to be done in understanding the contribution of PhoP-regulated genes in these processes. Of particular interest are secreted proteins (e.g., EsxG, EsxH, Mpt pilin, and MAB_0346) whose homologs in other mycobacteria have been shown to promote intracellular survival. Also intriguing is the finding among PhoP-regulated genes of a number of ORFs encoding putative polysaccharide and fatty acid-modifying enzymes that may participate in the cell surface makeup of MABS and thus modulate initial interactions with immune cells and downstream immune responses to infection. Defining in which (glyco)lipid, (lipo)polysaccharide, or glycoprotein biosynthetic pathway these genes may be involved is likely to reveal important species-specific strategies used by MABS to establish a chronic infection in the human lung.

## MATERIALS AND METHODS

### Strains and culture media

The reference strain *M. abscessus subsp. massiliense* CIP108297 was used as the wild-type parental strain throughout the study. The construction of *Mmas*Δ*phoPR* was described previously (5). For complementation, the WT *phoPR* operon and 130 bp of upstream DNA containing the promoter region were PCR-amplified from *Mmas* CIP108297 genomic DNA and ligated into pMV306-xylE, yielding pMV306::*phoPR* ^WT^. Complementation plasmids used for the production of mutated variants of PhoP (D71N and K224A) and/or PhoR (P77Q, T140K, and H258Q) in *Mmas*Δ*phoPR* were generated from pMV306::*phoPR* ^WT^ by site-directed mutagenesis (Genewiz). pMV306::*phoP* ^WT^, the plasmid used for complementation with *phoP* only, was generated by PCR-amplifying and cloning the *phoP* gene from *Mmas* CIP108297 into pMV306-xylE. The sequence of the primers used for PCR-amplifications is available upon request. *Mmas* strains were grown in Middlebrook 7H9 medium (Difco) supplemented with 10% albumin-dextrose-catalase supplements (BD Biosciences) and 0.05% Tween 80. The minimal base medium used for pH, phosphate, metals, nitrogen and carbon sources, nitric oxide, and hydrogen peroxide stress assays consisted of 0.085% NaCl, 50 µM FeCl_3_, 0.59 µM MnSO_4_, 3.5 µM ZnSO_4_, 4.5 µM CaCl_2_, 0.7 mM Na_2_HPO_4_, 1.8 mM KH_2_PO_4_, 20 mM L-Asparagine, and 0.05% tyloxapol in 50 mM MOPS buffer (for media at pH 7.0) or 50 mM MES buffer (for media at pH 5.7). Final pH was adjusted to the desired value with NaOH. When needed, glucose, glycerol, and pyruvate were each added to 10 mM final concentration. Oleic acid was used at 200 µM final concentration. Kanamycin (Kan; 200 µg/mL), hygromycin (Hyg; 1 mg/mL), or streptomycin (Str; 200 µg/mL) were added to the media preparations as needed.

### Luciferase-based monitoring of *phoP* expression

About 130 bp of the promoter region of *phoP* from *Mmas* CIP108297 was PCR-amplified (see Table S5 for primer sequences) and cloned into plasmid pMV306hsp + LuxG13 (Addgene plasmid #26161) (55) so as to replace the *hsp60* promoter driving the expression of the bacterial luciferase operon (*luxABCDE*). The resulting plasmid was introduced by electroporation into WT *Mmas* CIP108297 and *Mmas*Δ*phoPR*. For luciferase assays, 100 µL of *Mmas* cell cultures grown in different media was transferred to white 96-well plates with transparent bottom. The luminescence of the cells was measured on a Victor X plate reader using a preset protocol and the values were expressed as counts per second (CPS) and normalized to the OD_600 nm_ of the cultures.

### Metabolic labeling and analysis of total lipids

Radiolabeling of exponentially-growing whole *Mmas* strains (OD_600 nm_ = 0.5) with [1,2-^14^C]acetic acid (1 µCi/mL; specific activity, 54.3 Ci/mol, PerkinElmer) was performed in 7H9-ADC-Tween 80 medium (pH 7.0) or minimal medium containing pyruvate as the carbon source (pH 5.7) for 4 h at 37°C with shaking. Total lipids were extracted with a mixture of CHCl_3_:CH_3_OH, separated by thin-layer chromatography on aluminum-backed silica gel 60-precoated plates F254 (Merck) run in a variety of solvent systems (56), and finally imaged using a Sapphire Biomolecular Imager (Azure Biosystems).

### RNA extraction and RT-qPCR

*Mmas* cultures were grown to OD_600 nm_ = 0.2–0.3 in minimal medium (pH 7.0 or pH 5.7) with 10 mM glucose and 200 µM oleic acid as carbon sources, and RNA extracted using Direct-ZOL RNA miniprep kit (Zymo Research). cDNA was synthesized from RNA using SuperScript IV Reverse Transcriptase (Thermo Fisher) and qPCR was performed using the SsoAdvanced Universal SYBR Green Supermix (Bio-Rad) on a CFX96 Thermal Cycler (Bio-Rad). The target cDNA was normalized internally to the *sigA* cDNA levels in the same sample. PCR conditions: 98°C (30 s; enzyme activation), followed by 40 cycles of 98°C (10 s; denaturation) and 60°C (30 s; annealing/extension). Mock reactions (no reverse transcription) were done on each RNA sample to rule out DNA contamination. The sequences of the primers used to amplify *sigA*, *phoP*, and PhoP regulon genes are provided in Table S5. For RT-qPCR of Mmas-infected THP-1 macrophages, infections were established as described below and at the indicated time points, 1 mL TRIzol reagent (Thermo Fisher) was added to each well. Cells were scraped, and RNA extracted and processed as described above.

### RNAseq library preparation and data analysis

RNA-seq library preparation and data processing were conducted as described previously (53). Gene expression and differential expression analysis were completed in R (version 3.6.0) using DESeq2 (version 1.26.0) (57). Genes were identified as differentially expressed if they had a log_2_ fold change equal or greater than 1 and a Benjamini-Hochberg multiple testing correction adjusted *P* value of 0.05 or less. Venn diagrams were designed and analyzed using InteractiVenn (58).

### PhoP production and purification

Recombinant forms of PhoP from *Mmas* CIP108297, either WT or harboring D71N or K224A mutations, and bearing a hexahistidine tag at their N-terminal terminus were produced in *E. coli* BL21(DE3) using the pET14b expression system (Novagen, Madison, WI, USA). Following induction with 0.4 mM IPTG at 18°C in 2xYT broth overnight, cells were harvested, washed, and resuspended in lysis buffer consisting of 50 mM Tris-HCl (pH 7.5), 0.5 M NaCl, 10% glycerol, and protease inhibitor (0.2 mM 4-(2-aminoethyl)benzenesulfonyl fluoride hydrochloride). Cells were disrupted by sonication and insoluble material was removed by centrifugation for 30 min at 27,000 × *g*. The remaining lysate was applied to a HisTrap HP 5 mL column (Cytiva) equilibrated in lysis buffer containing 0.1 mg/mL phenylmethylsulfonyl fluoride (PMSF) and 0.16 mg/mL benzamidine. The column was then washed with 5 mL of buffer A (50 mM HEPES pH 7.3, 1.0 M NaCl, 10 mM imidazole, and 10% glycerol) and gradient eluted to 500 mM imidazole. Elution fractions containing the PhoP proteins were extensively dialyzed against buffer B (50 mM HEPES pH 7.3, 0.2 M NaCl, and 10% glycerol). Purity of the protein preparations was greater than 95% as judged by SDS–PAGE analysis and subsequent staining with Coomassie blue.

### Electrophoretic mobility shift assay

A 111 bp DNA probe containing the *phoP* promoter was PCR-amplified using fluorescently labeled primers IRDye700-phoPFw and IRDye700-phoPRv (see Table S5 for primer sequences). The binding reaction contained 50 mM HEPES pH = 7.3, 50 mM NaCl, 0.2 mg/mL BSA, 1 mM EDTA, 5% glycerol, 0.25% Tween 20, 2.5 mM DTT, 10 nM DNA probe, and different concentrations of recombinant purified PhoP in a final volume of 20 µL. Reactions were incubated for 1 h in the dark, after which 2 µL of 10× Orange loading dye (LI-COR) was added to each reaction, and 10 µL of the mixes was loaded on a Novex 6% DNA retardation gel (Thermo Fisher). The gel was run at 4°C in 0.5× TBE running buffer, removed from the cassette, and visualized using an Azure Sapphire imager.

PhoP phosphorylation was carried out by incubating the purified protein (1 mg/mL) with an equal volume of a 100 mM lithium potassium acetyl phosphate (Sigma) solution prepared in 50 mM HEPES pH 7.5, 20 mM MgCl_2_. The mixture was incubated at 37°C for 1 h and used directly in EMSA assays. To verify phosphorylation of the protein, samples were loaded on a SuperSep Phos-tag 7.5% gel (50 µmol/L) (Fujifilm), ran for 2 h in Tris-glycine SDS buffer and stained with Coomassie Blue.

### PhoR production and purification

The C-terminal cytosolic region (AA 180–503) of WT PhoR and mutants H258Q and T262A was PCR-amplified using primers phoR Fw and phoR Rv (Table S5). The PCR products were digested with NdeI and HindIII and cloned into the corresponding restriction sites of pET14b thereby adding an N-terminal hexahistidine tag to the constructs. Protein expression in *E. coli* BL21(DE3) cells harboring the plasmids was induced with 0.5 mM IPTG in LB medium for 16 h at room temperature. *E. coli* cells resuspended in lysis buffer (50 mM Tris-HCl, 150 mM NaCl, 10 mM MgCl_2_, and 10% glycerol, pH 7.5) containing 10 mM imidazole, 1 mM PMSF, and 0.1 µg/mL DNAseI were broken by bead beating and the lysate incubated with 200 µL HisPur Ni-NTA Resin (Thermo Fisher) for 2 h at 4°C. The beads were washed three times with lysis buffer containing 50 mM imidazole and eluted with lysis buffer containing increasing concentrations of imidazole (100, 150, 200, 250, and 300 mM). Eluted fractions were pooled and applied to Amicon Ultra 0.5 mL Centrifugal Filters 3K MWCO (Sigma) to concentrate the proteins and eliminate imidazole.

### Phosphatase assay

PhoP was phosphorylated with lithium potassium acetyl phosphate for 1 h as described above and passed through a Zeba Spin Desalting Column (Thermo Fisher) to eliminate residual acetyl phosphate. Phosphatase assay reactions contained 12.5 µg of phosphorylated PhoP, 30 µg of purified C-terminal catalytic domain from PhoR^WT^, PhoR^H258Q^, or PhoR^T262A^ and 3 mM ADP in 200 µL of reaction buffer (50 mM Tris-HCl, 150 mM NaCl, 10 mM MgCl_2_, and 10% glycerol, pH 7.5). Reactions were incubated at room temperature and, at the indicated timepoints, aliquots were withdrawn, mixed with 4× LDS loading buffer and immediately transferred on ice. Phos-tag gels (Fujifilm) were loaded with 10 µL of sample and run at 4°C for 2 h at 150 V. Immunoblots with anti-PhoP antibodies were performed as described below.

### Mouse polyclonal antibodies

Polyclonal antibodies to PhoP protein from *Mmas* CIP108297 were generated by immunizing BALB/c mice three times, 10 days apart, with 100 µg of purified recombinant PhoP protein. Mice were bled 14 days after the last immunization and the resulting serum was analyzed by ELISA to assess titers. For immunoblots, sera containing PhoP antibodies diluted 1:2,500 in 3% bovine serum albumin were incubated for 2 h with the blots. Goat anti-mouse IgG (whole molecule)–Peroxidase antibody (Sigma) diluted 1:5,000 was used as the secondary antibody. The Institutional Animal Care and Use Committee of Colorado State University approved all animal studies [IACUC protocol #1650; Assurance number D16-00345 (A3572-01)]. Studies were performed in accordance with recommendations of the Guide for the Care and Use of Laboratory Animals of the National Institutes of Health.

### Macrophage infections

THP-1 cells were grown in 24-well plates to ~90% confluence in RPMI and subsequently differentiated for 24 h with 15 ng/mL phorbol-12-myristate-13-acetate (PMA; Sigma). Well-dispersed cultures of *Mmas* strains (from frozen tittered stocks) diluted in 250 µL RPMI were used to infect the cells at a multiplicity of infection (MOI) of 1 for 2 h at 37°C. Cells were then washed three times with phosphate-buffered saline (PBS) and the wells were replenished with RPMI containing 250 µg/mL amikacin to kill extracellular bacteria. After an hour of incubation, cells were washed three more times with PBS and incubated in RPMI containing 50 µg/mL amikacin for the remainder of the experiment. At specified time points, viable intracellular *Mmas* was assessed by washing monocyte-derived macrophages two times with sterile PBS and then lysing the cells (in sterile water) and plating on 7H11 agar plates to enumerate colony forming units. All experiments were performed in triplicate biological replicates on at least two separate occasions and data represented as means ± SD with statistical significance determined using unpaired Student’s *t* test.

### Minimum inhibitory concentrations

*Mmas* CIP108297 was grown in 7H9-ADC-Tween 80 to OD_600 nm_=0.6–0.8 and diluted to OD_600 nm_= 0.01 in sterile water prior to being further diluted (1/50) in cation-adjusted Mueller Hinton II broth. Bacterial suspensions were added to 96-well plates containing appropriate antibiotic dilutions and incubated for 3 days at 37°C at which point wells were visually scanned for bacterial growth. Minimum inhibitory concentration is defined as the lowest concentration inhibiting growth.

## Data Availability

Raw sequence read files have been deposited in the Sequence Read Archive of the National Center for Biotechnology Information under accession numbers SRR24145896 to SRR24145884 (NCBI Bioproject no. PRJNA954523).

## SUPPLEMENTAL MATERIAL

Supplemental file 1 spectrum.01588-23-s0001.xlsx Table S1.

Supplemental file 2 spectrum.01588-23-s0002.xlsx Table S2.

Supplemental file 3 spectrum.01588-23-s0003.xlsx Table S3.

Supplemental file 4 spectrum.01588-23-s0004.pdf Tables S4 and S5 and Fig. S1 to S7.

ASM does not own the copyrights to Supplemental Material that may be linked to, or accessed through, an article. The authors have granted ASM a non-exclusive, world-wide license to publish the Supplemental Material files. Please contact the corresponding author directly for reuse.
