# Supplementary material for: Clinically relevant mutations in the PhoR sensor kinase of host-adapted Mycobacterium abscessus isolates impact response to acidic pH and virulence
Source: Microbiol Spectr. 2023 Oct 24;11(6):e01588-23. doi: 10.1128/spectrum.01588-23 (PMC10715180; doi:10.1128/spectrum.01588-23)

SUPPLEMENTAL MATERIALS

Clinically relevant mutations in the PhoR sensor kinase of host-adapted *Mycobacterium abscessus* isolates impact response to acidic pH and virulence

Juan M. Belardinelli[a], Divya Arora[b], Charlotte Avanzi[a], William H. Wheat[a], Josephine M. Bryant[b,c], John Spencer[a], Tom L. Blundell[d], Julian Parkhill[e,f], R. Andres Floto[b,c,g], Mary Jackson[a#]

From the [a]Mycobacteria Research Laboratories, Department of Microbiology, Immunology and Pathology, Colorado State University, Fort Collins, CO 80523-1682, USA; [b]Molecular Immunity Unit, University of Cambridge Department of Medicine, MRC-Laboratory of Molecular Biology, Cambridge, UK; [c]University of Cambridge Centre for AI in Medicine, Cambridge, UK; [d]Department of Biochemistry, University of Cambridge, Tennis Court Road, Cambridge CB2 1GA UK; [e]Wellcome Sanger Institute, Hinxton, UK; [f]Department of Veterinary Medicine, University of Cambridge, Cambridge, UK; [g]Cambridge Centre for Lung Infection, Papworth Hospital, Cambridge, UK

Supplemental Tables

Table S1 (RNAseq dataset; Excel file): Differentially expressed genes in *Mmas*Δ*phoPR* compared to *Mmas* WT at pH 5.7 and 7.0 (Log2-Fold Change (LFC) ≥ 1, or ≤ -1, padj<0.05). DE genes at both pHs are highlighted in yellow. Asterisks denote genes whose promoters harbor a putative PhoP binding site (DR1+DR2) (see Fig. S4).

Table S2 (RNAseq dataset; Excel file): Differentially expressed genes in *Mmas*Δ*phoPR* expressing *phoP*^{WT}, *phoPR*^{T140K} or *phoPR*^{H258Q} compared to *Mmas*Δ*phoPR* expressing *phoPR*^{WT} at pH 5.7 and 7.0 (LFC ≥ 1 or ≤ -1, padj<0.05). Asterisks denote genes whose promoters harbor a putative PhoP binding site (DR1+DR2) (see Fig. S4).

Table S3 (RNAseq dataset; Excel file): Differentially expressed genes in WT *Mmas*, *Mmas*Δ*phoPR*, *Mmas*Δ*phoPR* expressing *phoPR*^{WT}, *phoP*^{WT}, *phoPR*^{T140K} or *phoPR*^{H258Q} at pH 5.7 compared to pH 7.0 (LFC ≥ 1 or ≤ -1, padj<0.05).

Table S4: Minimum inhibitory concentrations (MICs) of antibiotics against WT *Mmas*, *Mmas*Δ*phoPR* and *Mmas*Δ*phoPR* complemented with *phoPR*^{WT}, *phoP*^{WT}, *phoPR*^{T140K}, *phoPR*^{P77Q} or *phoPR*^{H258Q}.

Table S5: List of primers used in this study.

Supplemental Figures

Figure S1: Kinetics of *phoP* expression in WT *Mmas* and *Mmas*Δ*phoPR* cultured in 7H9-ADC-Tween 80.

Figure S2: Effect of pH and carbon sources on *Mmas*Δ*phoPR* growth and *phoP* expression.

Figure S3: Growth of WT *Mmas* and *Mmas*Δ*phoPR* under different stress conditions.

Figure S4: Alignment of *Mtb* and *Mmas* PhoP and sequences of the DNA probes used in the EMSA assays.

Figure S5: Transcriptional profiles of WT *Mmas* and *Mmas*Δ*phoPR* expressing $phoP^{WT}$ only or different mutated forms of *phoPR* grown at pH 7.0 and 5.7.

Figure S6: Growth of *Mmas* WT and *Mmas*Δ*phoPR* expressing different mutated forms of PhoR in minimal medium at pH 5.7 and 7.0.

Figure S7: Lipid analysis of WT *Mmas* and *Mmas*Δ*phoPR* grown at neutral or acidic pH.

<u>Table S4</u>: Minimum inhibitory concentrations (MICs) of antibiotics against WT *Mmas*, *Mmas*Δ*phoPR* and *Mmas*Δ*phoPR* complemented with *phoPR*^{WT}, *phoP*^{WT}, *phoPR*^{T140K}, *phoPR*^{P77Q} or *phoPR*^{H258Q}.

MICs (in µg/ mL) were determined in Mueller-Hinton II medium using the resazurin microtiter assay as described in the Materials and Methods. AMK, amikacin; CLA, clarithromycin; CEF, cefoxitin; TIG, tigecycline; CFZ, clofazimine.

Strain	AMK	CLA	CEF	TIG	CFZ
Mmas WT	16	0.125	64	0.5	0.5
*Mmas*Δ*phoPR*	16	0.125	64	0.5	0.5
*Mmas*Δ*phoPR* + *phoPR*^{WT}	16	0.125	64	0.5	0.5
*Mmas*Δ*phoPR* + *phoP*^{WT}	16	0.125	32-64	0.5	0.25
*Mmas*Δ*phoPR* + *phoPR*^{T140K}	16	0.125	32-64	0.5	0.5
*Mmas*Δ*phoPR* + *phoPR*^{P77Q}	16	0.125	64	0.5	0.5
*Mmas*Δ*phoPR* + *phoPR*^{H258Q}	16	0.125	32-64	0.5	0.5

<u>Table S5</u>: List of primers used in this study.

Primer	Sequence (5' to 3')	Use
phoPR-Fw	CCAGTCTAGAGGCTCAACTGTAGGGCGACCTG	complementation with *phoPR* and *phoP* only
phoPR-Rv	GGAAAAGCTTGACGAGGCACAGGCCAAGATCAA	complementation with *phoPR*
phoP-Rv	AGCAAAGCTTACTGCTCTTCGCGCAGACACTCA	complementation with *phoP* only
PphoP-Fw	GCGTGCGGCCGCTCTAGAGG	*phoP* promoter fusion to *lux*
PphoP-Rv	TGCTGAATTCAGGTAATCTTCCCCCCAAAATTTAC	*phoP* promoter fusion to *lux*
phoP-Fw qPCR	CCGTGCTCAGTAAGCCCAAG	qPCR (*phoP*)
phoP-Rv qPCR	GCGCAGATACGAGACGTAGGA	qPCR (*phoP*)
sigA-Fw qPCR	CGTTCCTGGACCTGATTCAG	qPCR (*sigA*)
sigA-Rv qPCR	GTACGTCGAGAACTTGTAACCC	qPCR (*sigA*)
IRDye700-phoP Fw	GGCTCAACTGTAGGGCGACCTG	EMSA probe
IRDye700-phoP Rv	AGGTAATCTTCCCCCCAAAATTTAC	EMSA probe
phoP-Fw NdeI	CCGGCATATGGTGAGCATCACGACAGAGAC	*phoP* cloning into pET14b
phoP-Rv BamHI	AAGCGGATCCAGGCACAGGCCAAGATCAAGACT	*phoP* cloning into pET14b
phoR-Fw NdeI	TTGGCATATGCACCGCAGCCTGCGTCCGCT	*phoR-Cter* cloning into pET14b
phoR-Fw BamHI	CAAGGGATCCGAGGCACAGGCCAAGATCAAGA	*phoR-Cter* cloning into pET14b
phoR-Fw qPCR	GGGACAACTGGTGGAGGATC	qPCR (*phoR*)
phoR-Rv qPCR	CACCAGCAAATCGACCAACC	qPCR (*phoR*)
MAB0926-Fw qPCR	GCCTACTGCGATGTGGACTT	qPCR (*MAB_0926*)
MAB0926-Rv qPCR	AGTGTCCCGAAATCAGTGGC	qPCR (*MAB_0926*)
MAB1115-Fw qPCR	CGTACTCGTTCGGGATGCAT	qPCR (*MAB_1115*)
MAB1115-Rv qPCR	CGTTCCCCGTGGTGTAGATG	qPCR (*MAB_1115*)
MAB4531-Fw qPCR	GGTCTGGTGCTGTTGTTCCT	qPCR (*MAB_4531*)
MAB4531-Rv qPCR	GTCATCCCAGCTCCGGTG	qPCR (*MAB_4531*)

<u>Figure S1</u>: Kinetics of *phoP* expression in WT *Mmas* and *Mmas*Δ*phoPR* cultured in 7H9-ADC-Tween 80.

WT *Mmas* and *Mmas*Δ*phoPR* harboring a luciferase reporter gene under control of the *phoPmas* promoter (P*phoP-lux*) were grown in 7H9-ADC-Tween 80 at 37°C. At the indicated time points along the growth curve, samples were withdrawn and their luminescence (counts per second; CPS) measured and normalized to the OD_{600nm} of the cultures. The results presented are representative of two independent experiments.

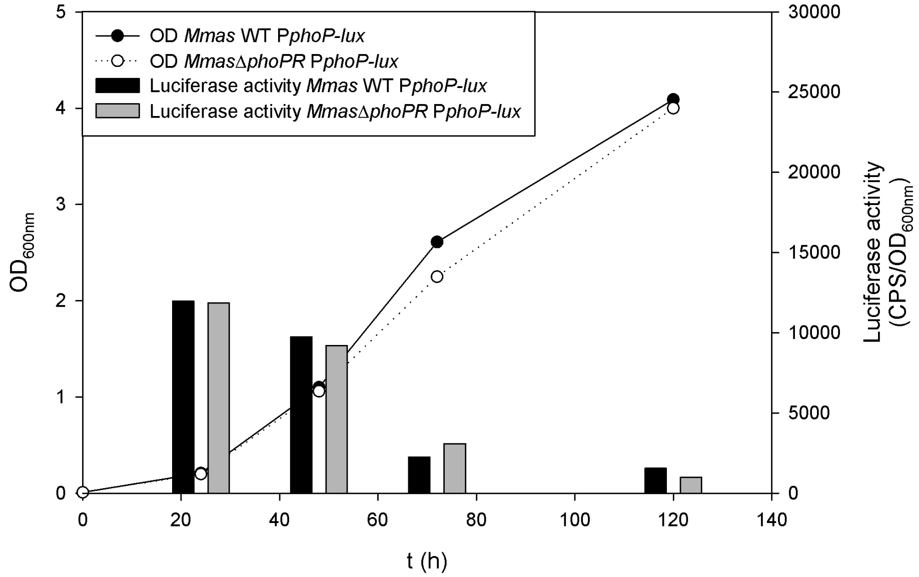

<u>Figure S2</u>: Effect of pH and carbon sources on *Mmas*Δ*phoPR* growth and *phoP* expression.
(A) *Mmas* WT and *Mmas*Δ*phoPR* were grown in minimal medium containing different carbon sources (10 mM glucose [Glu], 10 mM glycerol [Gly], 10 mM pyruvate [Pyr] or a combination of 10 mM glucose and 200 µM oleic acid [Glu + OA]) at neutral or acidic pH. Independent of the carbon source, the deletion of *phoPR* does not affect *in vitro* growth at low pH. The results are representative of two independent experiments.
(B) The luminescence of *Mmas* WT and *Mmas*Δ*phoPR* harboring the P*phoP-lux* reporter construct and cultured at described above was measured when cultures reached an OD_{600nm} ~ 0.2-0.3. Luciferase activity (CPS) is normalized to the OD_{600nm} of the cultures. Assays were performed in triplicate and are representative of at least two independent experiments. Asterisks denote statistically significant differences between pH 7.0 and 5.7 pursuant to the unpaired Student's *t*-test (****p*<0.0005).

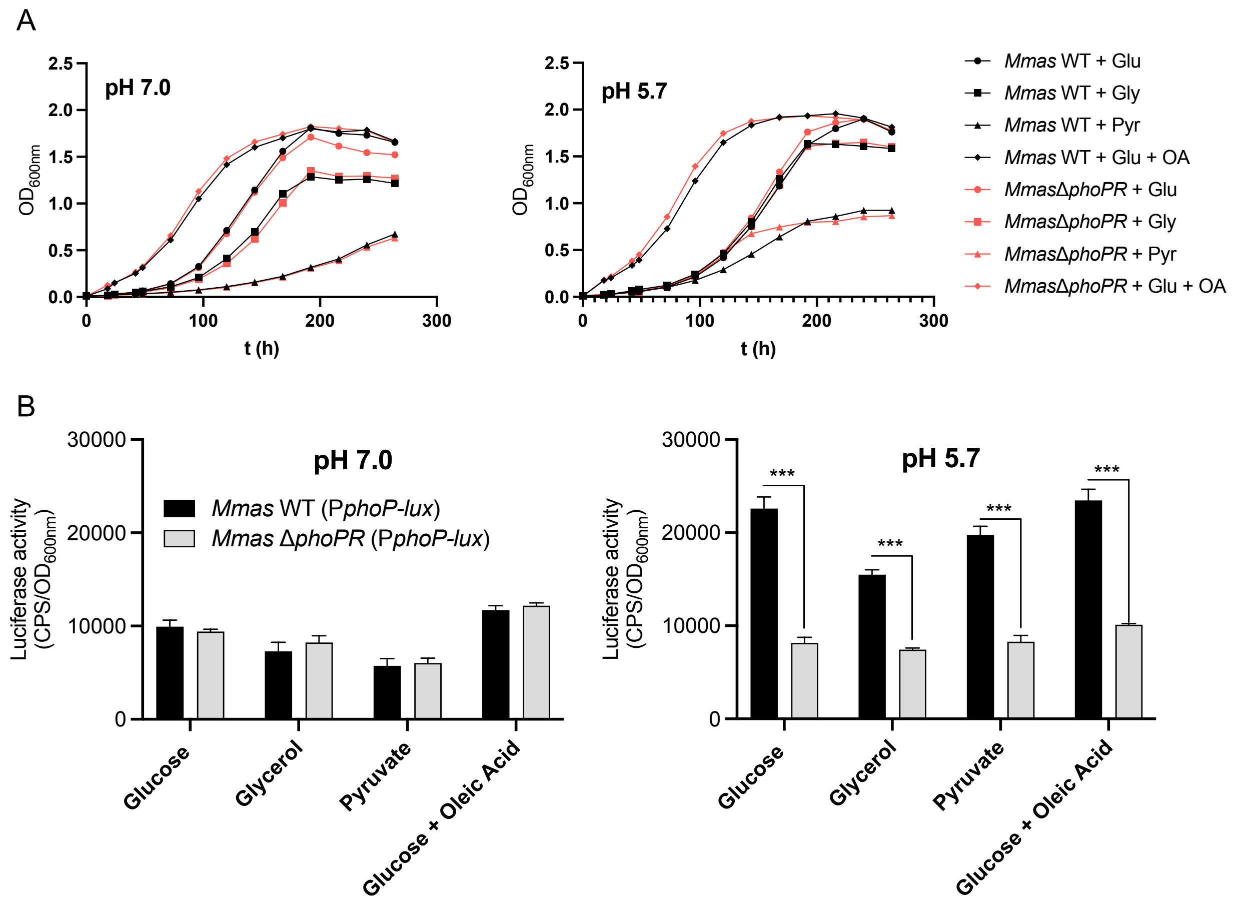

<u>Figure S3</u>: Growth of WT *Mmas* and *Mmas*Δ*phoPR* under different stress conditions.

WT *Mmas* WT and *Mmas*Δ*phoPR* were grown in minimal medium at pH 7.0 with 10 mM glucose and 200 μM oleic acid as carbon sources.

(A) The medium was supplemented with various constituents as indicated on the graphs to test the effects of a variety of stresses on the growth of *Mmas*Δ*phoPR*: magnesium concentration; NaCl concentration; potassium concentration; iron concentration, phosphate concentration or asparagine, nitrate, nitrite or ammonium as nitrogen sources. The results are representative of two independent experiments.

(B) Survival of *Mmas* WT and *Mmas*Δ*phoPR* under hypoxia (Wayne model). Cells were grown to exponential phase, diluted to OD_{600nm}=0.005 in Dubos medium and transferred to glass tubes (filled to 2/3 volume) with stir bars. Tubes were tightly capped and transferred to a magnetic stirrer plate at 37°C. After 0, 7, 14 or 21 days, three independent tubes were opened, cultures were diluted and plated on 7H11-ADC plates to enumerate CFUs. The arrow denotes the time at which cultures became hypoxic.

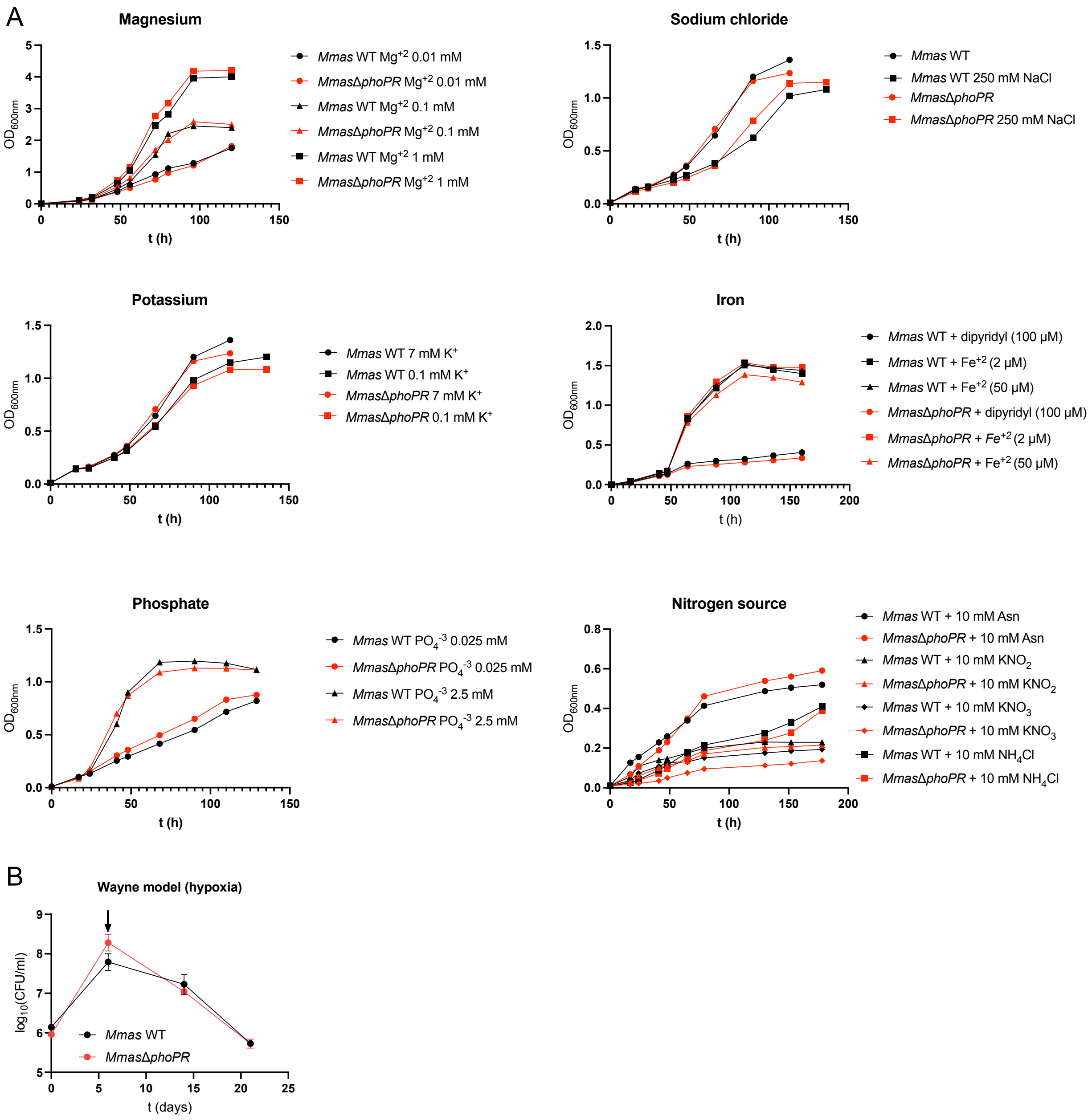
A
Magnesium
Mmas WT Mg+2 0.01 mM
MmasΔphoPR Mg+2 0.01 mM
Mmas WT Mg+2 0.1 mM
MmasΔphoPR Mg+2 0.1 mM
Mmas WT Mg+2 1 mM
MmasΔphoPR Mg+2 1 mM
OD600nm
t (h)
Sodium chloride
Mmas WT
Mmas WT 250 mM NaCl
MmasΔphoPR
MmasΔphoPR 250 mM NaCl
Potassium
Mmas WT 7 mM K+
Mmas WT 0.1 mM K+
MmasΔphoPR 7 mM K+
MmasΔphoPR 0.1 mM K+
Iron
Mmas WT + dipyridyl (100 µM)
Mmas WT + Fe+2 (2 µM)
Mmas WT + Fe+2 (50 µM)
MmasΔphoPR + dipyridyl (100 µM)
MmasΔphoPR + Fe+2 (2 µM)
MmasΔphoPR + Fe+2 (50 µM)
Phosphate
Mmas WT PO4-3 0.025 mM
MmasΔphoPR PO4-3 0.025 mM
Mmas WT PO4-3 2.5 mM
MmasΔphoPR PO4-3 2.5 mM
Nitrogen source
Mmas WT + 10 mM Asn
MmasΔphoPR + 10 mM Asn
Mmas WT + 10 mM KNO2
MmasΔphoPR + 10 mM KNO2
Mmas WT + 10 mM KNO3
MmasΔphoPR + 10 mM KNO3
Mmas WT + 10 mM NH4Cl
MmasΔphoPR + 10 mM NH4Cl
B
Wayne model (hypoxia)
log10(CFU/ml)
Mmas WT
MmasΔphoPR
t (days)

<u>Figure S4</u>: Alignment of *Mtb* and *Mmas* PhoP and sequences of the DNA probes used in the EMSA assays.

(A) Alignment of the *Mmas* CIP108297 and *M. tuberculosis* H37Rv PhoP proteins showing the high similarity between orthologs, in particular in the C-terminal region responsible for DNA binding. Identical residues are highlighted in red. The conserved Asp71 and Lys224 residues are in black boxes.

(B) Sequences of the DNA probes used in the EMSA assays shown in Figure 2B and 2C. The putative DR1 and DR2 binding sites (WT and mutated) are in bold letters.

A

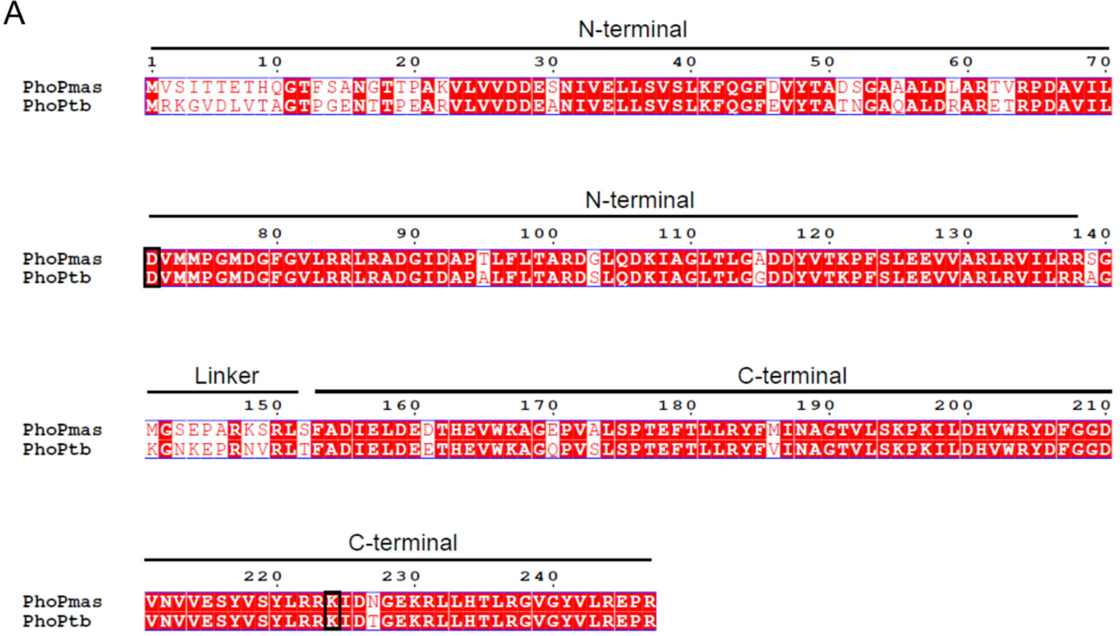

B

	DR1 DR2
PhoPmas promoter **WT**	TC**ACAGGAAGC**TC**ACAGTGAAG**ATCCAGCGCATCGTAAATTTTGGGGGGAAGATTACCTATG
PhoPmas promoter **DR1mut**	TC**GATCCGTCG**TC**ACAGTGAAG**ATCCAGCGCATCGTAAATTTTGGGGGGAAGATTACCTATG
PhoPmas promoter **DR2mut**	TC**ACAGGAAGC**TC**GGCTAACCT**ATCCAGCGCATCGTAAATTTTGGGGGGAAGATTACCTATG
PhoPmas promoter **DR1mut-DR2mut**	TC**GATCCGTCG**TC**GGCTAACCT**ATCCAGCGCATCGTAAATTTTGGGGGGAAGATTACCTATG

<u>Figure S5</u>: Transcriptional profiles of WT *Mmas* and *Mmas*Δ*phoPR* expressing *phoP*^{WT} only or different mutated forms of *phoPR* grown at pH 7.0 and 5.7.

Quantitative reverse transcription-PCR (RT-qPCR) (black) and RNA-seq (log_2 fold-change) (grey) show five differentially expressed genes at pH 7.0 (A) and pH 5.7 (B) compared to *Mmas*Δ*phoPR* expressing *phoPR*^{WT}: *phoP*, *phoR, MAB_0926* (putative glycosyltransferase family 2)*, MAB_1115* (hypothetical protein) and *MAB_4531* (CD225/dispanin family protein). Bacterial cultures were grown as described in Table 2. Ratios of *genes*/*sigA* mRNA are means ± standard deviations (n = 3 RNA extractions and RT-qPCR reactions).

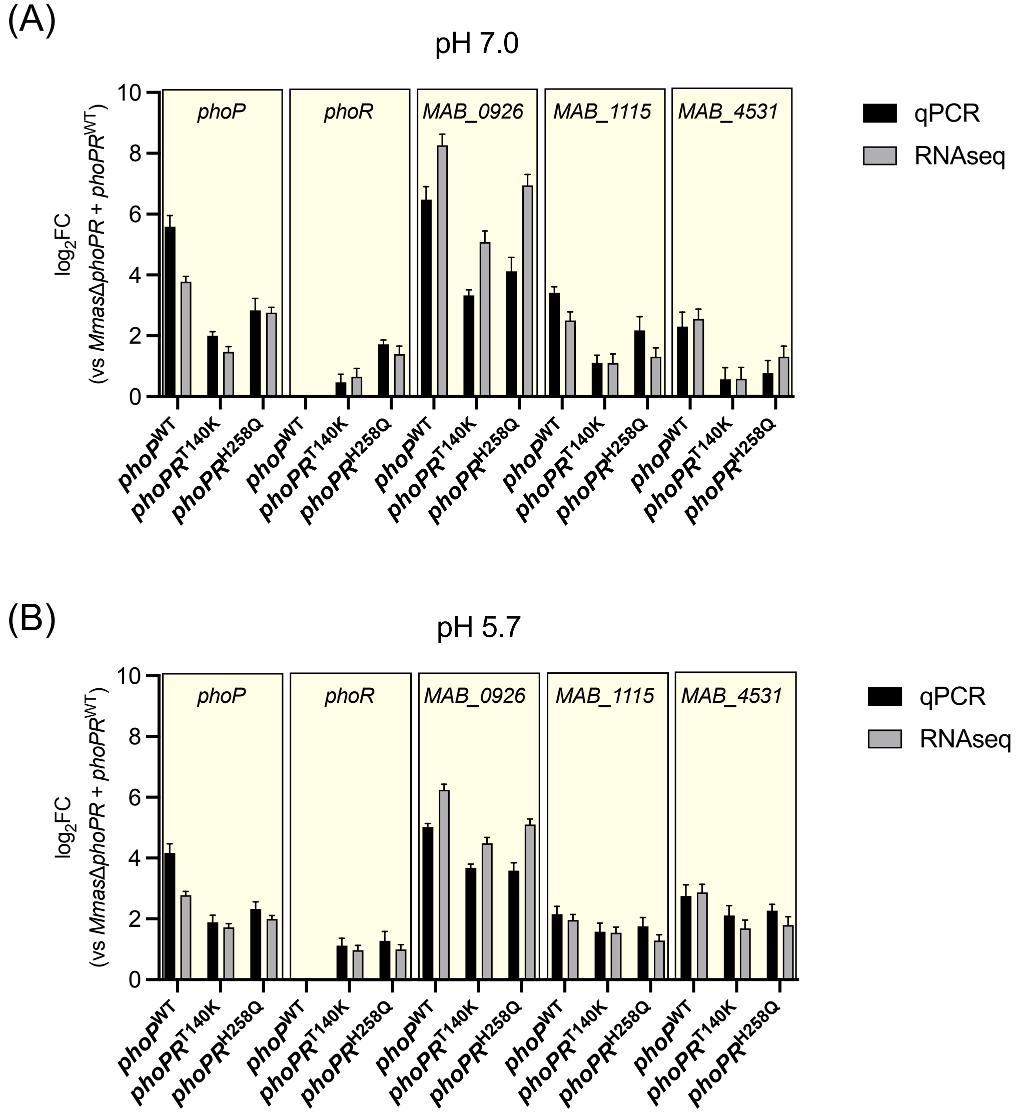

<u>Figure S6</u>: Growth of *Mmas* WT and *Mmas*Δ*phoPR* expressing different mutated forms of PhoR in minimal medium at pH 5.7 and 7.0.

The following strains are shown: *Mmas* WT, *Mmas*Δ*phoPR* and *Mmas*Δ*phoPR* expressing either *phoPR*^{WT}, *phoP*^{WT}, *phoPR*^{T140K} or *phoPR*^{H258Q}. 10 mM glucose and 200 µM oleic acid were used as carbon sources. The growth curves shown are representative of two independent experiments.

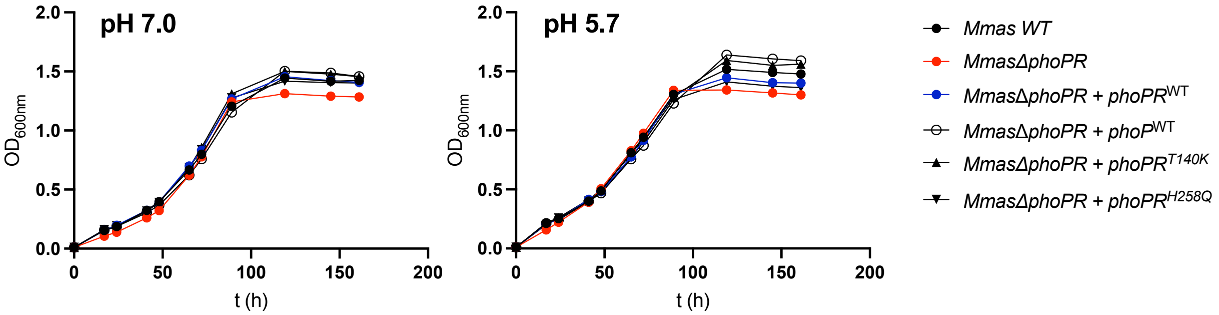

<u>Figure S7</u>: Lipid analysis of WT *Mmas* and *Mmas*Δ*phoPR* grown at neutral or acidic pH. Bacteria were grown at 37°C in 7H9-ADC-0.05% Tween 80 at pH 7.0 or in minimal medium at pH 5.7 with 10 mM pyruvate as the carbon source and labeled for 4 h with [1,2-^{14}C]-acetate. Total lipids extracted from WT *Mmas* and two independent clones of *Mmas*Δ*phoPR* were analyzed by thin-layer chromatography in two different solvent systems to reveal lipids of different polarities: (A) petroleum ether:diethyl ether (98:2 by vol., 3 developments); (B) chloroform:methanol:water (20:4:0.5 by vol., one development). TAG: triglycerides; TDM: trehalose dimycolates; TMM: trehalose monomycolates; PE: phosphatidylethanolamine; CL: cardiolipin; GPL: glycopeptidolipids.

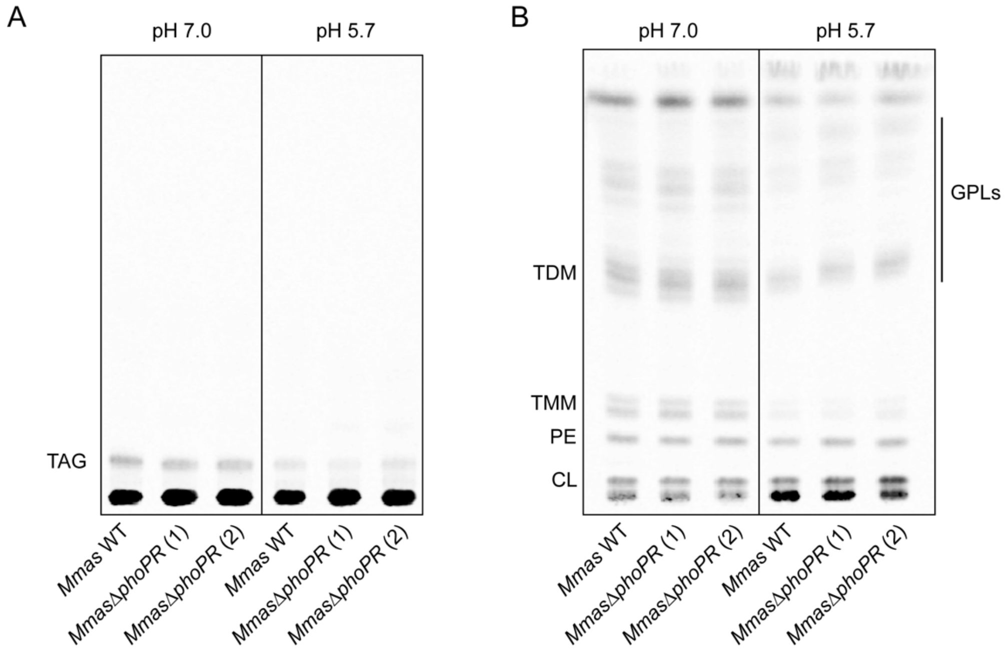

Supplement: Supplemental file 4 — Tables S4 and S5 and Fig. S1 to S7. [file spectrum.01588-23-s0004.pdf]
